# Role of wetlands in reducing structural loss is highly dependent on characteristics of storms and local wetland and structure conditions

**DOI:** 10.1038/s41598-021-84701-z

**Published:** 2021-03-04

**Authors:** Y. Peter Sheng, Adail A. Rivera-Nieves, Ruizhi Zou, Vladimir A. Paramygin

**Affiliations:** Coastal and Oceanographic Engineering Program, Engineering School of Sustainable Infrastructure and Environment, University of Florida, Gainesville, FL 32607 USA

**Keywords:** Ecology, Environmental sciences, Natural hazards, Ocean sciences

## Abstract

Coastal communities in New Jersey (NJ), New York (NY), and Connecticut (CT) sustained huge structural loss during Sandy in 2012. We present a comprehensive science-based study to assess the role of coastal wetlands in buffering surge and wave in the tri-state by considering Sandy, a hypothetical Black Swan (BS) storm, and the 1% annual chance flood and wave event. Model simulations were conducted with and without existing coastal wetlands, using a dynamically coupled surge-wave model with two types of coastal wetlands. Simulated surge and wave for Sandy were verified with data at numerous stations. Structural loss estimated using real property data and latest damage functions agreed well with loss payout data. Results show that, on zip-code scale, the relative structural loss varies significantly with the percent wetland cover, the at-risk structural value, and the average wave crest height. Reduction in structural loss by coastal wetlands was low in Sandy, modest in the BS storm, and significant in the 1% annual chance flood and wave event. NJ wetlands helped to avoid 8%, 26%, 52% loss during Sandy, BS storm, and 1% event, respectively. This regression model can be used for wetland restoration planning to further reduce structural loss in coastal communities.

## Introduction

On October 29 of 2012, Superstorm Sandy made landfall near Brigantine, NJ with 36 m/s maximum sustained wind and minimum pressure of 945 mbar. It caused widespread flooding in the US mid-Atlantic coast, specifically in the states of New Jersey (NJ), New York (NY), and Connecticut (CT), due to its massive size, and the coincidence of the surge with the astronomical spring high tide^1^. New water level records were set at National Oceanic and Atmospheric Administration (NOAA) stations along the tri-state coasts. According to the Federal Emergency Management Agency (FEMA) Modeling Task Force Hurricane Sandy Impact Analysis, the tristate coasts were the most impacted coasts during Sandy. We focus on this large region due to its large population (~ 20 million) and Gross Metropolitan Product (GMP) of ~ $2 trillion^2^.

A large amount of data was obtained during Sandy, including the storm tide and wave data at NOAA and the National Data Buoy Center (NDBC) stations, water level and barometric pressure data at the 224 United States Geological Survey (USGS) sensors for continuous monitoring along the U.S. Atlantic coast, and an extensive survey of High Water Marks (HWMs) following Sandy’s landfall^3^. This large dataset provides a unique opportunity for scientific understanding and model verification of the coastal surge-wave-tide dynamics and the role of wetlands in buffering flood and wave during extreme storm events in this large region.

Previous observational and numerical assessment studies show that coastal wetlands can reduce coastal flooding^4–7^. Wamsley et al.^8^ showed measured surge reduction rates across marsh transects in the range of 1.7 cm/km to 25 cm/km and numerically simulated reduction rates of about 2 cm/km to 17 cm/km on the Louisiana coast. By using a 2D model and observed data, they concluded that wetlands have the capacity of reducing surge but is very dependent on the bathymetry, wetland structure, and storm characteristics^8–10^. Sheng et al.^11^, using a 3D vegetation-resolving surge model, showed the reduction of total inundation volume in a coastal area during hurricanes as a function of hurricane characteristics and marsh distribution and structure. Sheng and Zou^12^, using the vegetation-resolving surge-wave model, Curvilinear Hydrodynamic in 3D Storm Surge Modeling System (CH3D-SSMS), showed a significant reduction of total inundation volume by mangroves in Miami-Dade County, Florida during Hurricane Andrew in 1992.

Recent studies showed contradictory findings on the value of coastal wetlands in reducing structure loss along the New Jersey coast during Superstorm Sandy. Narayan et al.^13^ (hereafter referred to as NAR17), using a 2D hydrodynamic model and an industry-based flood and loss model to quantify the wetland value in terms of structure losses, found that, on the zip-code level, wetlands helped to significantly avoid an average of 27% of structure loss in NJ and up to 139% in south NJ^14^. Paradoxically, their estimated avoided loss is only 3% over the entire NJ, suggesting wetland had little impact. The percent avoided loss was calculated as the ratio of the avoided loss (difference between the loss without wetland and the loss with wetland) divided by the loss with wetland. In zip-code with low loss, however, the percent avoided loss became misleadingly high. Moreover, their assessed loss was not verified with FEMA National Flood Insurance Program (NFIP) payout data, making it impossible to validate their assessment. Their south NJ analysis was based on idealized (instead of actual) structure data and the effect of wetland on reducing wave and wave-induced loss, which can be comparable to the flood loss, was not explicitly addressed. Lathrop et al.^15^ (hereafter referred to as LAT19), on the other hand, used NFIP loss payout data with actual structure and wetland data for south NJ constructed a regression model to relate the structure losses to several factors and found that wetland had little effect on structure loss. Neither study was able to convincingly relate the actual loss to the various contributing factors including wetland type and coverage, wave and surge, and actual structural data. To resolve the conundrum created by these two studies, we present a comprehensive study to elucidate the role of wetlands in affecting flood, wave, and structure loss during three very different flood and wave events.

A typical “dynamics-based” assessment (as opposed to the “regression-based” assessment of LAT19) on the value of wetland for flood reduction is done by simulating the flooding and wave caused by a hurricane, first with and then without the wetland^11–13,15–17^. Loss associated with the flood and wave is then calculated with and without the wetland^13,16–18^. However, there are large uncertainties associated with many aspects of this “dynamics-based” assessment: accuracy of the simulated hurricane wind fields used for the surge-wave model, model parameterization of surge-wave dynamics and vegetation processes, availability of wind-surge-wave data during hurricanes for verification, accurate representation of the coastal wetland and properties, inclusion of flood and wave losses, and parameterization of loss processes in the loss model, etc^19–21^. Importantly, flood and wave both contribute to structure loss, and wave-induced loss can exceed flood-induced loss for some storms. Therefore, unless the assessment includes detailed verification of the simulated wind and surge-wave, as well as estimated structure loss, the assessed loss reduction by wetland, could be highly uncertain and may be useless for local resilience planning efforts.

The 2D approach using Manning coefficients to include the bottom friction and vegetation-induced friction is the go-to methodology in most hurricane loss assessment studies, as in NAR17, although it has been shown that the Manning coefficient is a function of vegetation structure and flow, thus making its parameterization very complex and uncertain^22,23^. Extensive water level and flow data are needed to tune the Manning coefficient which is a function of space and time. Nevertheless, while the 3D model is preferred over the 2D model, we used the 2D version of CH3D-SSMS for this study due to lack of detailed coastal wetland structure data along the tristate coasts. The simulated surge and wave during Sandy were verified with extensive field data, while the estimated residential structure losses were verified with FEMA NFIP loss payouts^24^. Additionally, we assessed the role of coastal wetlands in reducing loss during an extreme “Black Swan” storm (see “Methods” section for the rationale behind the Black Swan storm) which made landfall near Staten Island, NY, and an ensemble of storms along the tristate coasts. Real structure data, as opposed to idealized structural data^13^, were used in the loss analysis for Sandy, the Black Swan storm, and ensemble of storms. Importantly, this study explicitly simulated the surge and wave as dynamically coupled processes and calculated the loss by both flood and wave, while NAR17 simulated the surge only and considered the wave effect “implicitly”^13^. While flood and wave analyses were done for the tristate region, the loss analysis was done for NJ and parcel-specific loss analysis results were aggregated into zip-code scale and state scale for in-depth analysis. Loss analysis for NY and CT could not be conducted due to unavailability of structural data.

## Results

### Extensive hydrodynamic model validation

To provide a comprehensive validation of the modeled surge-tide-wave dynamics during Sandy, this study made use of all available field data from an extensive network of coastal water level gauges and wave buoys in the study region, as well as hundreds of HWMs and many rapid deployment surge sensors (SI Fig. S1). The good agreements between simulated and observed water levels and waves were quantified in terms of the root-mean-square error (RMSE) and correlation coefficient (CORR).

#### Storm tide

Available data included those from hundreds of permanent and temporary surge sensors from NOAA (SI Fig. S2), Hudson River Environmental Conditions Observing System (HRECOS), and USGS. Statistics (SI Table S1) showed excellent agreement between the time series of simulated and measured storm tide data. The averaged RMSE at USGS temporary sites was 0.20 m and the averaged CORR was 0.94. CH3D-SSMS successfully reproduced the surge and tides with high confidence in not only the open ocean but also the complex estuarine system during Sandy (SI Fig. S3) with a maximum coastal storm tide at NY Bight of 3.36 m and NJ coast of 3.27 m.

#### High water marks and inundation

A total of 526 out of 653 independent HWM locations were located within the study region, most of which clustered in NJ and NY coastal areas. 83.7% (440 out of 526) of HWMs were captured by the CH3D-SSMS model grid and compared with the surveyed values. The model results had 0.33 m RMSE and 0.87 CORR (SI Fig. S4). When only “good” and “excellent” HWMs (rated by USGS) were used, the RMSE dropped to 0.30 m, and CORR increased to 0.90. The noticeable data disagreements (Fig. SI 4) were caused by the inconsistency of surveyed data.

#### Wave

During Sandy, four wave-buoys within the domain recorded the wave data: two at the apex of NY Bight, and another two in Long Island Sound (LIS). The significant wave height $H_{sig}$ and peak wave period $T_{p}$ simulated by the Simulating Waves Nearshore (SWAN) model in the CH3D-SSMS were compared with measured data and Wave Watch III (WW3) operational run results (SI Fig. S5)^25,26^. SWAN more accurately captured the evolution of $H_{sig}$ in NY Bight and LIS. Maximum significant wave height over land was as high as 2.17 m at NY Bight and 2.60 m along NJ coast.

### Impact of wetland on surge and wave during super storm sandy

To estimate the value of the coastal wetlands in reducing flood, we calculated the following four metrics for inundation: the Average Inundation Height ($AIH$), the Maximum Inundation Height ($MIH$), the Total Inundation Area ($TIA$), and the Total Inundation Volume ($TIV$) with and without wetlands. The $TIA$ and $TIV$ are defined as

1 $$ TIA =  \iint_{{{\text{Landward}}\;{\text{area}}}} {dxdy} , $$

2 $$ TIV =  \iint_{{{\text{Landward}}\;{\text{area}}}}  [H_{max} \left( {x,y} \right) - H_{0} \left( {x,y} \right)]dxdy, $$

where $H_{max} \left( {x,y} \right)$ and $H_{0} \left( {x,y} \right)$ are the maximum water level and the land elevation at land cells $\left( {x,y} \right)$, respectively^11,12^. The wave analysis was carried out by calculating the average wave height ($AWH$), the maximum wave height ($MWH$), and the total wave energy ($TWE$) which is defined as

3 $$ TWE =  \iint_{{{\text{Landward}}\;{\text{area}}}}   [\frac{1}{8}\rho_{w} g{ }\left( {H_{{rms\,max{ }}} } \right)^{2} ]dxdy, $$

where $H_{rms max}$ is the maximum root-mean-square wave height over the flooded land. The wave energy, instead of wave height, is more directly related to the wave-induced structure loss.

**Figure 1:**
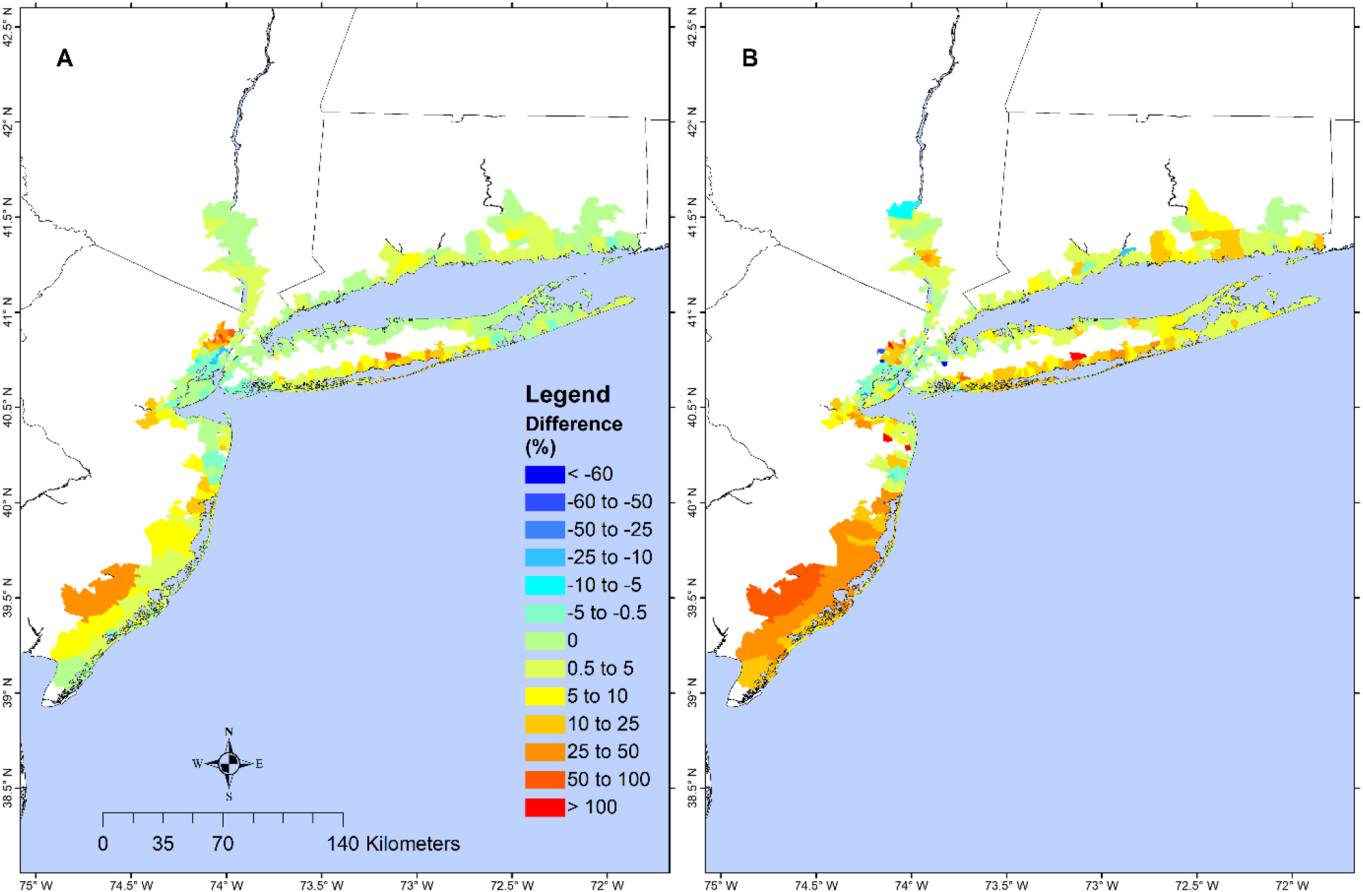
Zip-code resolution wetland’s effect on $TIV$ and $TWE$ during Sandy in 2012. Map showing zip-code resolution avoidance in (**A**) $TIV$ and (**B**) $TWE$ during Sandy without wetlands, as a percentage of those for the with-wetland scenario. Dark red values show zip-code with the most wetland benefit while dark blue areas have the least wetland benefit. Negative values indicate that the presence of wetland would increase $TIV$/$TWE$ and positive values indicate that wetland would lower $TIV$/$TWE$. The map is produced using ESRI ArcGIS Pro 2.7 (https://www.esri.com/en-us/arcgis/products/arcgis-pro/overview).

We define the relative inundation reduction ($RIR$) as the difference in $TIV$ (value without wetland minus value with wetland), divided by the $TIV$ with wetland. The relative wave reduction ($RWR$) is defined accordingly using the $TWE$. The inundation and wave analysis were carried out at the regional level (SI Table S2) and zip-code resolution (Figs. 1, S6, S7).

If the wetlands were absent during Sandy, the $RIR$ of the entire model domain would have been 4% and the $RWR$ would have been 19%. Breaking the entire model domain into 6 regions (Table S2): South NJ (SNJ), Central NJ (CNJ), North NJ (NNJ), CT, Long Island (LI), and Mainland NY (MNY), it was found that the $RIR$ during sandy was low (< 10%) for all regions, while the $RWR$ was moderate for CNJ and SNJ (10–25%) where the wetland cover was high, and low for the other regions. Although CT ranked 3rd in wetland cover, its $RWR$ ranked 4th during Sandy due to Sandy’s landfall location and the sheltering of waves provided by LI which ranked 3rd.

Model simulations show that, during Sandy, if the wetland in the entire region were completely made up of woody wetland, TIV and TWE would decrease by 9.1% and 12.4%, respectively, while increase by 1.2% and 2.2%, respectively, if the wetland were composed of marsh only. $RIR$ and $RWR$ for the all-marsh wetland are 6.5% and 19.1%, respectively, while those for the all-woody-wetland are 17.6% and 36.8%, respectively. These results demonstrate that the woody wetland is much more effective than the marsh in buffering flood and wave.

### Benefit of wetland on surge and wave during a Black Swan (BS) storm

We consider a rare “Black Swan” (BS) storm (with a 0.0034 annual frequency vs. 0.0014 for Sandy^27^) which made landfall in Staten Island with a storm surge that surpassed the maximum surge during Sandy with maximum coastal values of 7.17 m at NY Bight and 4.51 m at NJ coast of (Fig. S8). On the other hand, maximum significant wave heights over land were not greater than those of Sandy: 1.99 m at the NY Bight and 2.12 m on the NJ coast, but average wave height was higher than during Sandy (Fig. S8). During the Black Swan storm, wetlands created the biggest $RIR$ and $RWR$. MNY and NNJ experienced significant $RIR$, while the remaining regions experienced low $RIR$. In contrast to Sandy, in which CT, MNY, and NNJ had less $RWR$, for the Black Swan storm these regions experienced high (> 50%) $RWR$, while LI. CNJ and SNJ had significant (~ 25–50%) $RWR$. The regions with the highest $RIR$ and $RWR$ were closest to the storm landfall location.

### Benefit of wetland on surge and wave for the 1% annual chance flood

The maximum 1% annual chance maximum flood elevation (Fig. S9) was 5.15 m for NY Bight while 5.34 m for the NJ coast. The 1% annual chance maximum wave height (Fig. S9) overland is 2.24 m at NY Bight and 1.90 m at NJ coast. All the regions had moderate $$RIR$$ except for SNJ which had a significant $$RIR$$ due to highest (25.4%) wetland cover (see Table S2) and NNJ experienced significant $$RWR$$ while the remaining regions had moderate values. CT ranked 2nd in $$RIR $$ although it has less wetland cover than CNJ because the mostly woody wetlands in CT are more effective in buffering storm surge than the marshes in NJ and NY. On the other hand, CT ranked 3rd in $$RWR$$ due to the blocking of offshore wave energy by LI. $$RIR$$ and $$RWR$$ are found to be functions of storm characteristics, wetland type, and cover, and local conditions. Fig. S10 shows the percent wetland cover, RIR (relative TIV reduction), and RWR (relative wave energy reduction) in six regions (New York, North New Jersey, Long Island, Connecticut, Central New Jersey, and South New Jersey) during 1% annual chance events. As the wetland cover increases from less than 5% (NY, NNJ, and LI) to more than 10% in CNJ and SNJ, RIR and RWR generally increase, showing the increasing role of wetland in reducing inundation and wave. Relative reduction in inundation and wave energy are modest, between 10 and 30%. NNJ has properties behind the relatively sparse marsh, followed by woody wetland which protects properties behind them. Connecticut has less wetland than Central Jersey, but the mostly woody wetland is more effective in reducing flood and wave.

### Benefit of wetlands on reducing residential structure loss

**Figure 2:**
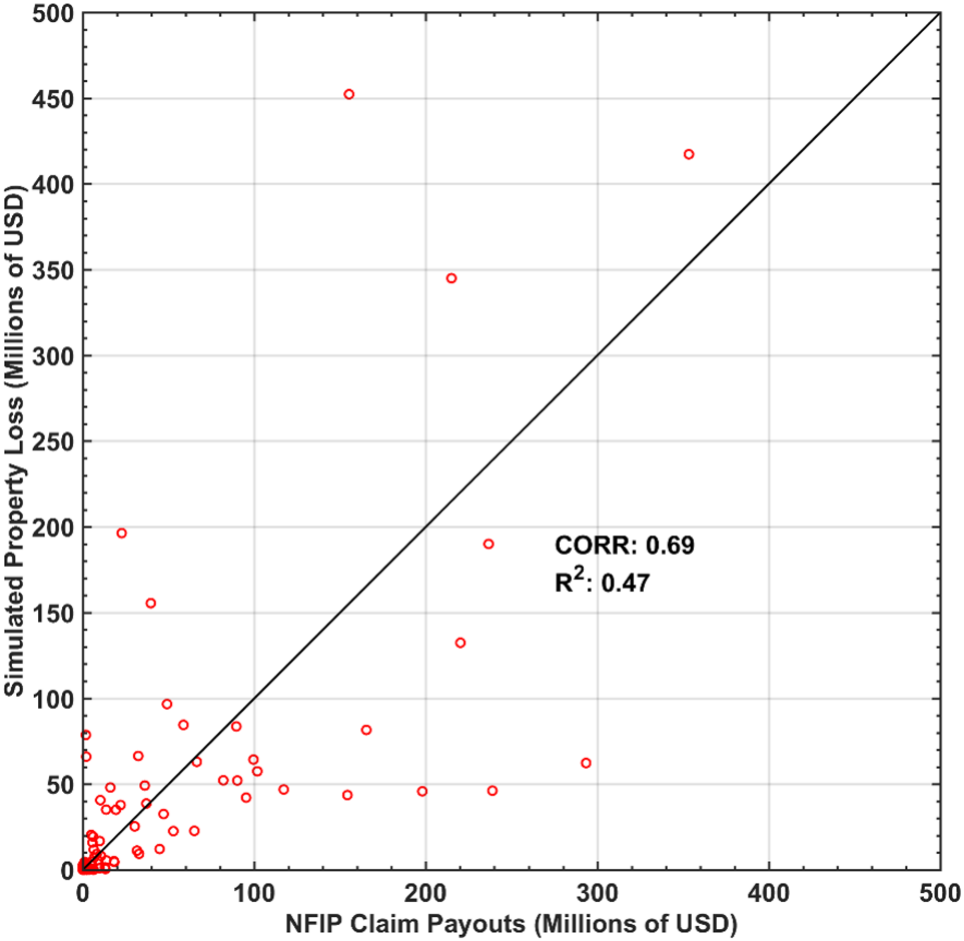
Economic model validation at zip-code resolution. Simulated losses during Sandy in NJ using the USACE damage functions versus FEMA NFIP payouts. Results were aggregated by zip-code and the corresponding correlation coefficient is 0.69 (R^2^ = 0.47). Validation use transformed structure loss ($PL_{T}$) instead of the structure loss ($PL$). The figure is produced using MATLAB R2020 (https://www.mathworks.com/).

The monetary loss of residential structures was estimated using the simulated inundation and wave results while employing damage functions from the United States Army Corps of Engineer (USACE) North Atlantic Comprehensive Coastal Study (NACCS) and was validated using the NFIP building loss payouts aggregated by zip-code^21,24^. Direct simulation of wave-induced damage requires understanding and calculation of wave loads on structures using a depth- and phase-resolving model, which is beyond the capability of the models used in this study. Therefore, in this paper, we did not directly simulate wave-induced damage, but are accounting for wave-induced change in total water level which results in increase in estimated damage based on depth-damage functions. Overall, 96 coastal zip-codes in the state of NJ were used to validate the estimated loss. The model showed a correlation coefficient (CORR = 0.69) between simulated structure losses and NFIP payouts (Fig. 2). In NJ, as of 2019, the total NFIP payout was $3.9 billion USD, in comparison to the estimated total structure loss of $3.6 billion USD (SI Table S3), with an absolute error of 7.7%. This good agreement, plus the good agreement between the simulated and observed surge and wave reported earlier, confirms the validity of our “dynamics-based” loss assessment.

**Figure 3:**
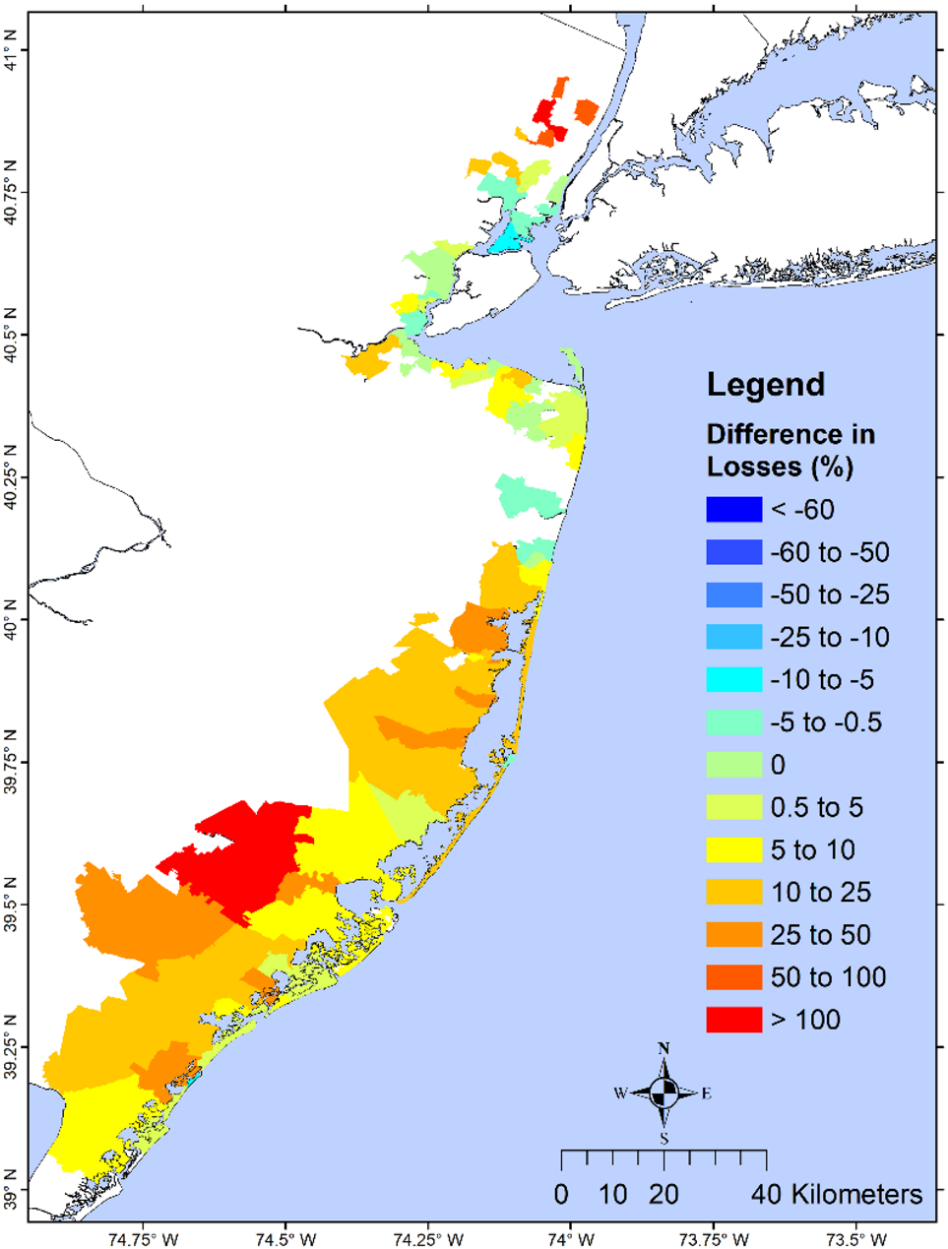
Percent structural loss reduction during Sandy. Map showing zip-code resolution avoided loss (difference in loss without wetlands and loss with wetlands) during Sandy, as a percentage of the loss of the with-wetland scenario. Dark red values show zip-code with the highest wetland benefit, while dark blue areas have the least benefit. Negative values indicate that the presence of wetland would increase structural losses and positive values indicate that wetland would lower the structural losses. The results are shown for the NJ coastal zip-codes affected by Sandy. This study shows that the percent avoided loss in this figure does not always represent the actual wetland value for loss reduction because areas with few structures and losses could give misleadingly high values of percent avoided loss, as shown in south NJ. The primary purpose is for comparison with a similar figure in NAR17. The map is produced using ESRI ArcGIS Pro 2.7 (https://www.esri.com/en-us/arcgis/products/arcgis-pro/overview).

**Figure 4:**
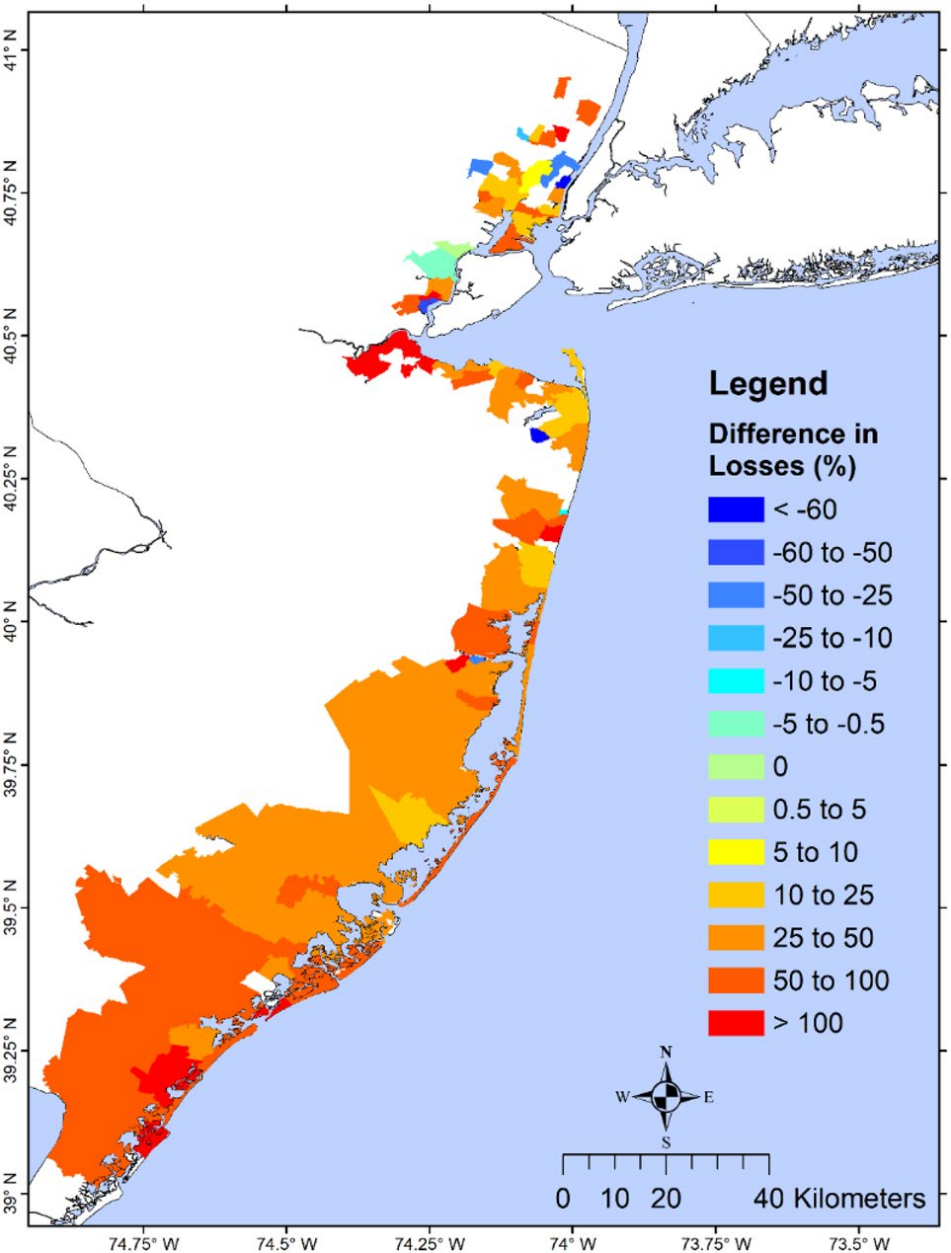
Effect of wetlands on structural losses over zip code scale during the 1% annual event. Map showing zip code resolution difference in losses if the wetlands were absent, as a percentage of the wetland present scenario. Dark red values show zip code with the highest benefit of having wetlands while dark blue areas show the least benefited area. Negative values indicate that the presence of wetland would increase structural losses and positive values indicate that wetland would lower the structural losses. The map is produced using ESRI ArcGIS Pro 2.7 (https://www.esri.com/en-us/arcgis/products/arcgis-pro/overview).

We define the structural loss reduction ($SLR$) as the structural loss without wetland minus the structural loss with wetland, and the relative structural loss reduction ($RSLR$) as the ratio between $SLR$ and the loss with wetland. A state-level analysis of structure loss in NJ showed a $RSLR$ of 8.5%, 26.0%, and 52.3% for Sandy, BS storm, and 1% chance flood/wave, respectively (Table S3). Analysis of losses due to flood and wave indicated that for Sandy and the 1% event, most of the loss came from flood, while most of the loss in the BS storm came from waves. Avoided wave-induced loss was comparable to the avoided flood-induced loss during the BS storm and the 1% event, but much higher during Sandy, suggesting that NJ wetlands are more effective in reducing wave-induced loss vs. flood-induced loss. Results from the zip-code scale analysis during Sandy (Fig. 3) showed less variability: for most north NJ, $RSLR$ ranged from 10 to 100% except for those along the Hudson River that had small increased loss (negative avoided loss). Most zip-codes in SNJ had $RSLR$ between 5% and more than 100%. $RSLR$ during the BS storm (Fig. S11) was more notable: ~ 50–100% for north NJ, 0–100% for central NJ. The 1% flood event (Fig. 4) showed an average RSLR > 25% for NJ.

The above results showed that the value of coastal wetlands for flood/wave protection varies significantly with the storm. While wetlands may be more effective in reducing wave loss in some storms but flood loss in other storms, they may be ineffective in extreme storms. The 1% annual chance flood and wave event, which resulted from an ensemble of many less extreme but more frequent storms^28^, provides a more reasonable integrated scenario for the loss analysis. This is similar to the preferred use of the 1% flood map, instead of the flood map associated with a single design storm, for assessing the flood risk in any coastal region.

**Figure 5:**
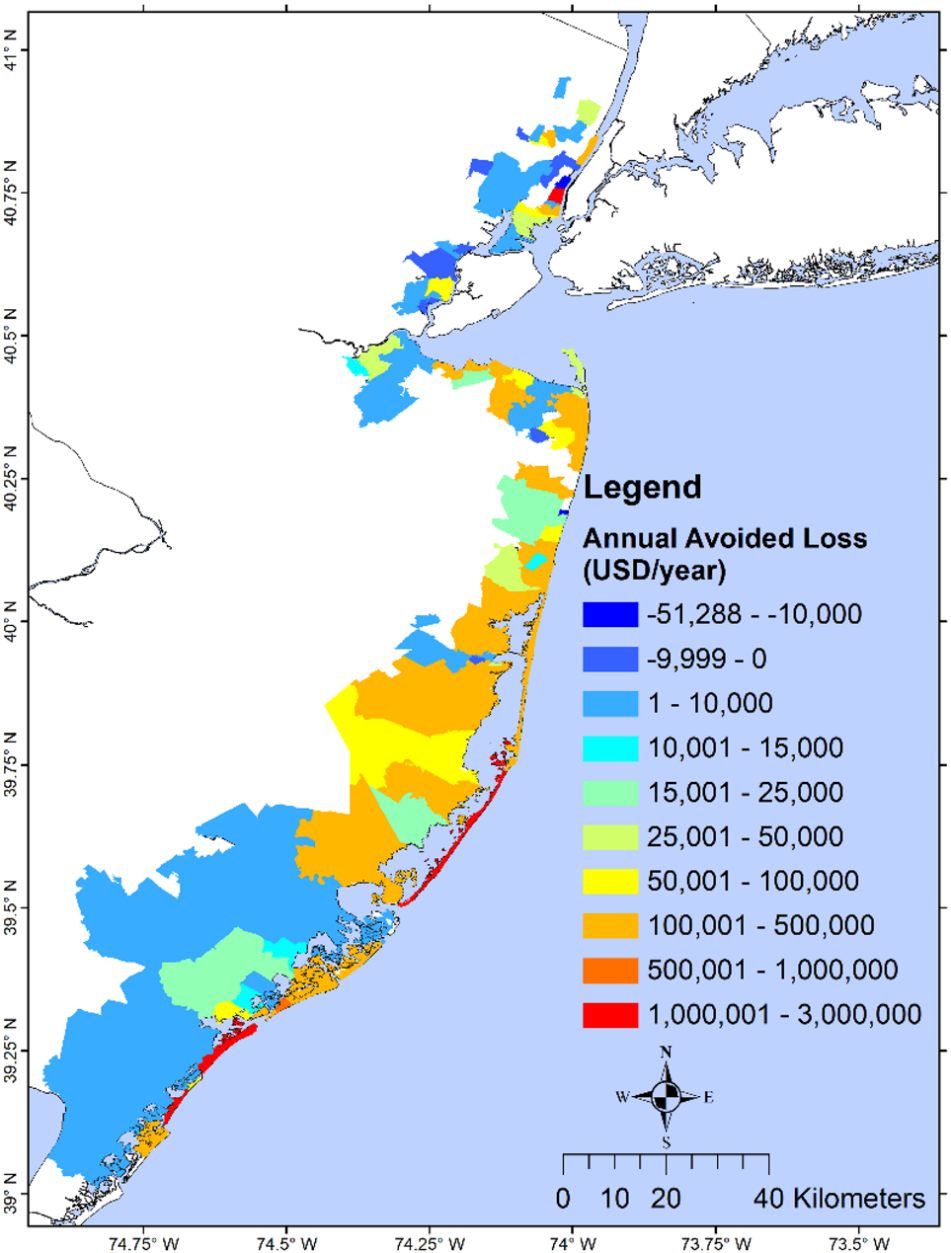
Wetland annual avoided losses for all NJ zip-codes. Map showing zip-code resolution avoided losses. Annual avoided loss is calculated as the difference in losses for the without wetland and with wetland scenarios using the 1% annual chance flood and wave map and then dividing it by 100 years. Dark blue values show zip-code with the lowest wetland value/benefit, while red areas have the highest wetland value/benefit. Negative values indicate that the presence of wetlands would increase structural loss and positive values indicate that wetlands would lower structural loss. Negative values are relatively small while only increasing losses by ~ $10 thousand per year. The map is produced using ESRI ArcGIS Pro 2.7 (https://www.esri.com/en-us/arcgis/products/arcgis-pro/overview).

As shown in Fig. 5, in zip-codes with larger wetland coverage area in SNJ, wetlands could only prevent < $25,000 annual loss (loss in the 1% event divided by 100), while wetlands in zip-codes with medium wetland coverage area in central NJ could prevent > $60,000 annual loss. On the other hand, wetlands in north NJ zip-codes with smaller wetland coverage area would increase the loss. The annual loss in most of the zip-codes is < $10,000 except in one zip-code where the annual loss is ~ $50,000.

**Figure 6:**
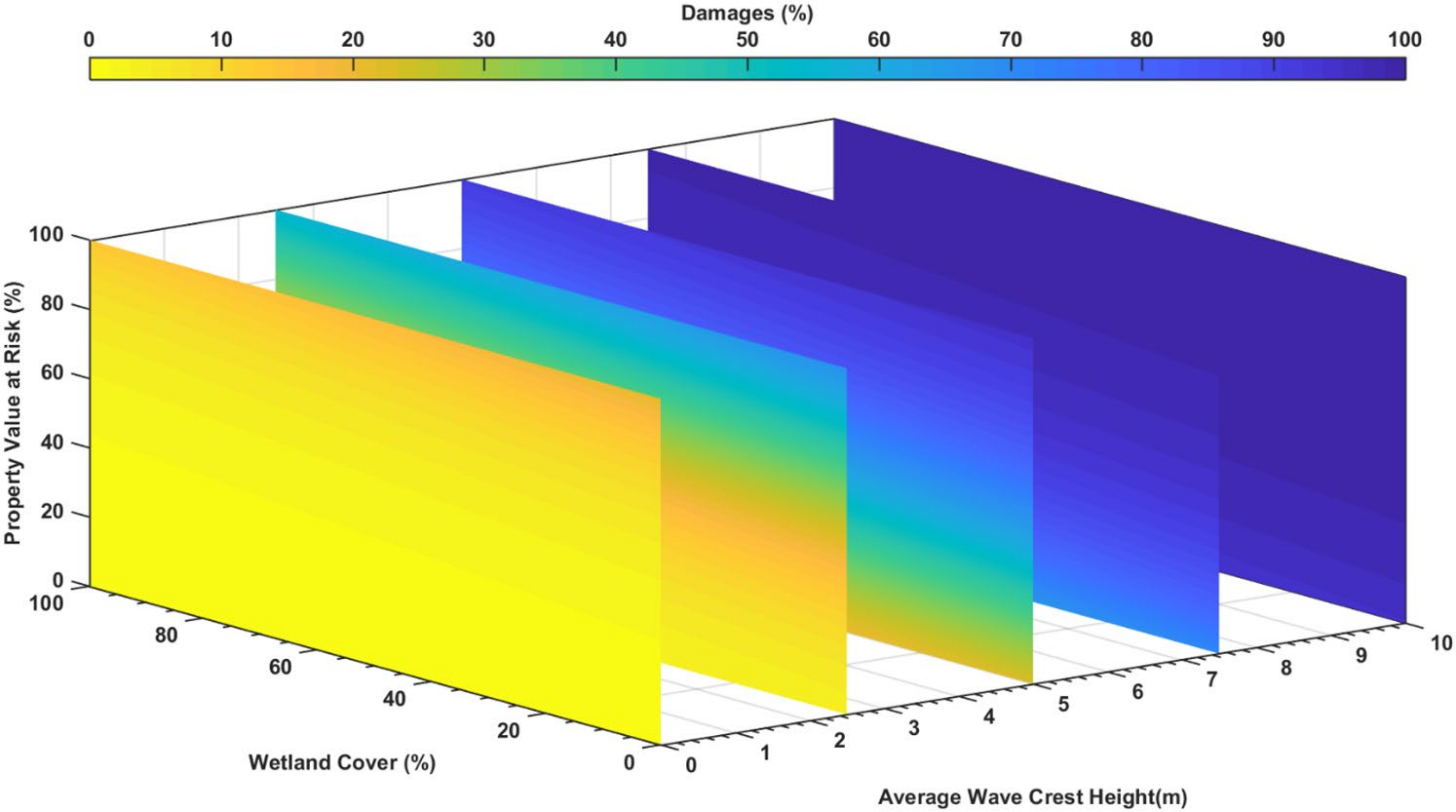
Zip-code structural loss regression model. A generalized regression model using a normal distribution with a logit link function. Constructed using the Structure Value at Risk, Wetland Cover, and Average Wave Crest Height as predictors to estimate structural losses in zip-codes (R^2^ = 0.75, p < 0.001). Coupled with a Rapid Forecast and Mapping System^33^ being developed for the region, this regression model can be used to forecast potential structural loss during an approaching hurricane. It can also be used to predict the potential loss in any hypothetical hurricane, or to plan wetland restoration to reduce loss in any specific zip code. The figure is produced using MATLAB R2020 (https://www.mathworks.com/).

To explain the significant spatial variation in $SLR$ by wetlands, we developed a generalized linear regression model using the normal distribution with the logit link function, which was fitted with data for all coastal zip-codes during three flood events and with and without wetlands. Percent wetland cover, percent at-risk structural value, and average wave crest height in the zip-code were used as predictors to estimate the percent structural loss (PSL = structural loss value divided by at-risk structural value) in the zip-code (Table 1). For Sandy, the regression model had a significant correlation (R^2^ = 0.75, p < 0.001). For the BS storm and the 1% annual chance flood, there was a strong correlation with R^2^ = 0.87 and R^2^ = 0.84, respectively, both with p < 0.001. Using the three storm scenarios, each with and without wetland, the regression fit (Fig. 6) had a significant correlation with R^2^ = 0.75 and p < 0.001.

**Table 1:** Zip-code damage regression model estimates.

	Sandy	Black Swan	1% annual chance	All
**Estimated coefficients**				
Intercept	− 4.5960	− 4.5626	− 4.6232	− 5.3659
	(0.1663, 6.14E−68)	(0.2007, 2.16E−54)	(0.1330, 5.29E−90)	(0.1295, 2.83E−176)
Wetland cover	− 0.3750	− 0.4936	− 0.3759	− 0.5553
	(0.17477, 0.0332)	(0.2440, 0.0446)	(0.1627, 0.0218)	(0.1487, 0.0002)
Total structure value at risk	3.1588	4.7737	3.5424	4.0563
	(0.1526, 2.93E−50)	(0.1813, 3.94E−63)	(0.1206, 4.94E−77)	(0.1182, 2.15E−142)
Average wave crest height	0.5809	0.5226	0.31778	0.83418
	(0.0845, 9.02E−11)	(0.0673, 6.27E−13)	(0.0623, 7.46E−07)	(0.0431, 7.58E−65)
N	191	181	218	590
R-squared	0.7487	0.8726	0.8359	0.7505
p-value	7.82E−56	6.36E−79	1.11E−83	3.52E−176

The first value inside parenthesis represents the standard error and the second value in parenthesis represents p-values.

## Discussion

In this paper, we presented the benefits of wetlands for reducing storm surge, waves, and structural losses during Hurricane Sandy. Recent papers on this topic, such as NAR17 and LAT19, focused on the effects of wetlands on reducing structure loss due to storm surge but did not include the effect of wetlands on reducing wave-induced structural loss^13,15^. Estimated loss in NAR17 was not compared to NFIP payouts. LAT19 pointed out that vegetation has a dampening effect on wave energy, but waves were not included in their empirical NFIP pay-out model due to lack of data^15^. NAR17 did not include the wave in their hydrodynamic model explicitly^13^, although it is known that wave setup could contribute up to 20% of the peak surge^29^ and wave can introduce additional loss. Here, we showed how valuable these wetlands are in terms of reducing wave energy which could damage structures and even cause deaths. Wetland effect on attenuating storm surge has been widely studied and the attenuation effect increases with the wetland thickness, density, and height^11^. Our results showed that regions with thicker, denser, and taller wetland cover reduced more $TIV$ and $TWE$ during Superstorm Sandy. Regions with highest wetland cover, hence more $TIV$ and $TWE$ reduction, were found in SNJ, CT, LI, CNJ, NNJ and MNY, in descending order.

LAT19 observed that the extent of wetland on Barnegat-Great Bay did not have enough spatial extent and height to dissipate the Superstorm Sandy surge^15^. This is consistent with our finding that wetland dissipated $$TIV$$ by − 0.5% to 0.5%. Like NAR17, we found that landward zip-codes benefited from the surge attenuation at coastal zip-codes^13^. Moreover, we found that wave attenuation at coastal zip-codes helped to reduce wave energy at landward zip-codes. Our study showed notable $$TWE$$ dissipation in SNJ where the landward zip-codes experienced a cumulative reduction in surge and wave which translated into less structural loss (Fig. 3). For example, in zip-code 08330 (Hamilton Township), wetland helped to avoid 41% of structural loss, far less than the 139% reported in NAR17^13^. In contrast, we found that zip-code 08226 (Ocean City) had only a ~ 3% loss avoidance. The annual avoided losses for zip-code 08330 and 08226 were ~ $11,000 and ~ $3.6 million, respectively. This showed that percent avoided loss is not a good index for characterizing the wetland value for protection against storm surge and waves. Zip-code with low total structural value and low avoided loss, such as 08330, was estimated by NAR17 to have a misleadingly high value of percent avoided losses.

Our estimated loss compared reasonably well with the NFIP loss payouts. The squared correlation coefficient of 0.47 is much higher than the value of 0.184 of LAT19 who found no evidence that increasing marsh buffer distance would reduce the amount of NFIP payouts for the nine south NJ townships^15^. LAT19’s suggestion that NFIP payouts will increase by $4.05 per foot of marsh width was not supported by our dynamics-based economic analysis^15^.

Our economic analysis slightly under-estimated the total NFIP payouts, probably because of some conservative assumptions. Our hydrodynamic model did not consider major rivers in NJ and did not include precipitation. For simplicity, we used the 2D version of CH3D-SSMS with the Manning’s n coefficient representing the friction by vegetation, land, and structures, which likely overestimated the friction effect and reduced the loss. During Sandy, marsh stem height—usually lower than 30 cm in south NJ according to the 2012 land cover database of the NJ Department of Environmental Protection (NJDEP)—was much lower than the storm surge, therefore the actual marsh benefits were probably somewhat lower than that represented by the 2D model^30^. The effect of wetland structure, as well as marsh distribution, on flood and wave dissipation, could be better represented by using a 3D vegetation resolving model, e.g., Sheng et al.^11^, if detailed wetland data were available. Our structural loss model did not include unavailable basement data which could have increased the losses. The Manning’s n used in our simulation was 0.045 which is smaller than the value of 0.068 for rigid vegetation^31^ but very close to the value of 0.049 for flexible vegetation when the empirical findings of Chapman et al.^32^ were used to adjust the bulk drag coefficient in Luhar and Nepf^31^. If the *Spartina-*dominant marsh were replaced by a tall and dense *Phragmites-*dominant marsh*, *Manning’s n would increase and result in less structural loss.

During the BS storm, NJ coast experienced a slightly higher surge but much higher wave, both significantly buffered by the wetlands. Hence, the removal of wetlands would significantly increase the flood, wave, and loss along the NJ coast than during Sandy. This showed that wetlands’ buffering capacity depends significantly on the wetland structure/distribution, the characteristics of the hurricane, and the specific location of interest. To assess the overall buffering capacity of tidal wetlands over a large region, an ensemble of storms should be considered instead of 1–2 specific storms. To generate the 1% flood and wave event, 300+ “optimal storms” were generated with the JPM-OS method and the CH3D-SWAN model based on the ensemble of storms generated by a statistical hurricane model^26^. Flood and wave associated with these optimal storms are being used to develop a Rapid Forecast and Mapping System (RFMS)^33^ for NJ/NY/CT region, which can be used to predict real-time surge and wave including wetland effects during any hypothetical storm.

The regression analysis shows that wetlands are more effective in reducing losses when the average wave crest height is lower, which confirms that wetlands’ buffering capacity is more effective for low-intensity and more frequent storms^16^. Loss in a zip-code increased with the at-risk structural value and the average wave crest height but decreased with percent wetland cover. Zip-codes with higher structural value benefited more from wetland protection. Without any structure behind a wetland, the ecosystem service value of the wetland for flood protection is zero. The regression model developed in this study can be used to estimate the impact of changing wetland coverage on loss, e.g., doubling the wetland coverage in every NJ zip-code would decrease the average loss in Sandy from 4 to 3%.

Although coastal wetlands were found to reduce structural loss during Sandy, their percent loss reduction was found to be very modest (− 10% to + 50% for most except one zip-code) compared to 22–139%, reported by NAR17 in South NJ. At Himilton Township, NAR17 reported a dramatic 139% but LAT19 reported a slight increase. The over-estimation of wetlands’ buffering capacity by NAR17 possibly resulted from the uncertainties and lack of verification of their flood and loss analysis, not explicitly accounting for waves which contribute significantly to loss, and the use of percent avoided loss as the sole indicator for wetland value. No attempt was made to explain the effect of various contributing factors on the estimated losses.

**Figure 7:**
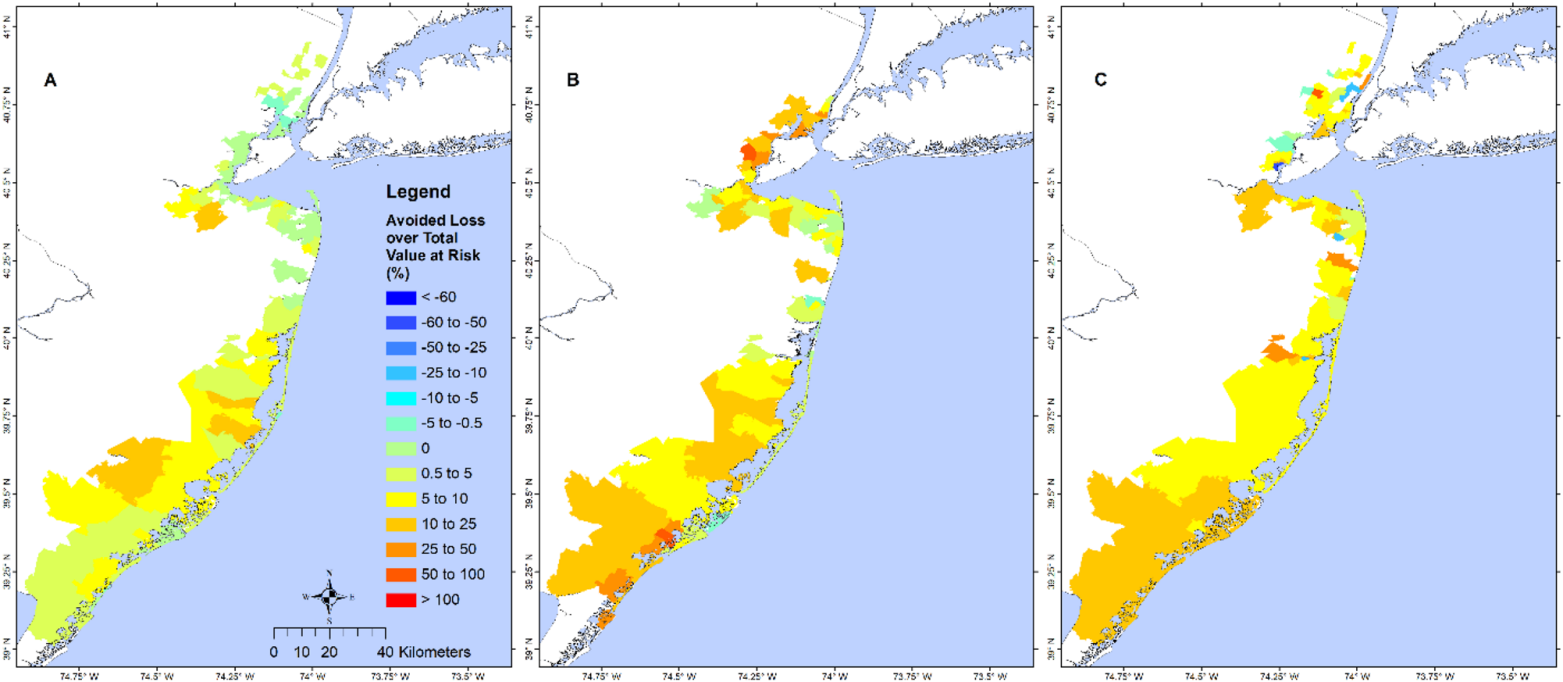
Wetland value at zip-code level. Map showing zip-code resolution avoided losses during (**A**) Sandy, (**B**) BS storm, and (**C**) 1% annual flood and wave, as a percentage of the total at-risk property value of the without wetlands scenario. Dark red values show zip-code with the highest wetland value while dark blue areas show the least wetland value. Negative values indicate that the presence of wetland would increase structural losses and positive values indicate that wetland would lower the structural losses. These maps show that wetland values for NJ are mostly between low (< 10%) and modest (10–25%). These maps, along with the wetland value regression model developed in this study, can be used by local planners to develop resiliency and restoration plans to increase the wetland coverage area and enhance the wetland value. Maps for NY and CT are unavailable due to unavailability of structural data there. The map is produced using ESRI ArcGIS Pro 2.7 (https://www.esri.com/en-us/arcgis/products/arcgis-pro/overview).

This study showed that the percent structural loss (PSL) varies significantly across a coastal region depending on percent wetland cover, at-risk structural value, and storm characteristics^11^ (represented by average wave crest height). As shown in Fig. 7, the PSL for NJ is between low (< 10%) and moderate (< 25%). These maps, together with the regression model can be used to develop wetland restoration plans (e.g., increasing wetland coverage area) to reduce structural loss in the future. Adding the regression model to the RFMS will enable forecasting of structural loss during a hurricane.

Strong winds during storms can directly damage coastal wetlands, resulting in reduced buffering capacity of wetlands for coastal protection. Further studies are needed to fully understand the complex interactions among wetlands (with different type, structure, density, and coverage area, etc.), storm surge and wave, erosion, wind, and sea-level rise (SLR). With 1–2 m of SLR projected by 2100, tristate tidal marshes (with a height of 0.3–1 m) can be easily overwhelmed and lose their buffering capacity^34^. The approach presented in this study can be expanded to develop and assess wetland restoration plans to mitigate future flood risk in the twenty-first century.

## Methods

The role of coastal wetland in reducing coastal flood is analyzed by determining (a) the surge peak and extent, (b) the significant wave height and peak wave energy, and (c) the resulting structural losses at all flooded locations during three flooding events (Sandy, “Black Swan” storm, and the 1% annual chance flood and wave event). The surge and significant wave height were estimated using a coupled surge-wave modeling system CH3D-SSMS and the structural loss was estimated using the USACE depth damage functions^19^.

### Hydrodynamic modeling

The coupled surge-wave model is based on CH3D-SSMS^29,35,36^. CH3D-SSMS couples 2D/3D storm surge and wave models of coastal and basin scales using a non-orthogonal curvilinear grid for the horizontal directions and a terrain-following sigma coordinate in the vertical direction for an accurate representation of estuaries and coastal waters. In the coastal region, the surge model CH3D is dynamically coupled to the wave model SWAN^25,37,38^. The governing modeling equations and surge-wave coupling mechanisms of CH3D-SSMS were well documented in Sheng et al.^29^.

The study domain is represented by a high-resolution curvilinear grid which consist of 1461 by 2182 cells with 40–50 m resolution in NYC and about 20 m in the low-lying land area, such as lower-Manhattan, to resolve the local complex geographic features. The grid domain covers the coasts of New Jersey, New York, and Connecticut. It also includes the Hudson River from the New York Harbor to the Federal Dam at Troy. It extends from the continental shelf to elevations that are higher than the possible extent of flooding by storm surge. The grid in CT and NY reach elevations that are greater than 200 m and in NJ it is expanded 40+ km inland or reaching elevations that are greater than 10 m. The model’s bathymetry and topography were generated using NOAA’s National Centers for Environmental Information (NCEI) bathymetric and topographic telescoping digital elevation models (DEMs) of the U.S. Atlantic Coast impacted by Hurricane Sandy. This DEM resolution varies from 1/9 arc-sec (3.4 m) in the coastal zone to 3 arc-sec (90 m) in the offshore. For the locations where NCEI’s DEM was not available, the 1/9 arc-sec DEMs from the U.S. Geological Survey (USGS) National Elevation Dataset (NED) was used for land elevations, while NCEI’s Coastal Relief Model (CRM) was used for the water cells. The Estuary Bathymetry data from the New York State Department of Environmental Conservation (NYSDEC) was used in the Hudson River between Newburgh and Troy. These elevations were interpolated to the grid cells.

For this study, a 2D version of CH3D-SSMS was implemented with the use of Manning’s friction coefficient (n) to account for the friction produced by vegetation and other land cover types. Higher values of Manning’s n imply greater resistance to flow due to higher horizontal shear stress throughout the water column. Land Cover types were obtained from the USGS 2011 National Land Cover Database (NLCD) which have a resolution of 30 m^39^. Manning’s n values were obtained from the land cover classes of the USGS NLCD based on the conversion table from Mattocks and Forbes^40^. According to this, the coastal wetlands represented by emergent herbaceous and woody wetlands have Manning’s n values of 0.045 and 0.1, respectively (Fig. S12).

#### Superstorm Sandy

The southern and eastern open ocean boundaries of the model are forced with linearly added predicted tides and storm surge elevation. The tidal constituents used in the model are M2, N2, K1, S2, O1, K2, and Q1 and they are obtained through the Advanced Circulation Model (ADCIRC) Tidal Dataset (version ec2001)^41^. The surge boundary conditions for CH3D-SSMS were provided by an ADCIRC large-scale model to incorporate the remote effects of storm surges^9,10^. Offshore wave conditions came from NOAA’s WW3 and river discharge data from the USGS. Two major rivers were included: the Hudson River at Green Island, NY (USGS 01358000), and Connecticut River at Thompsonville, CT (USGS 01184000). Two high-resolution wind fields, computed by the Regional Atmospheric Modeling System (RAMS), were applied as the meteorological forcing. A wind field that covers a smaller spatial area with a spatial resolution of 6 km and a temporal resolution of 15 min from Weatherflow, Inc. was merged with a larger spatial area wind field with a spatial resolution of 4 km and a temporal resolution of 1 h. These wind fields were also used by Wang et al.^42^ successfully to simulate storm surge during Sandy.

#### A “Black Swan” storm

The Black Swan (BS) storm was designed as an extreme scenario of a Cat-3+ hurricane such that winds of Category 1 hurricane intensity would be felt at the Village of Piermont, approximately 40 km upstream of NYC along the Hudson River, after the landfall at Staten Island (Fig. S13). This storm would create an extreme event in NJ because it moved northward very near the NJ shoreline. The wind field was generated using the Holland wind model^43^ with a pressure deficit of 45 mbar, a radius to the maximum wind of 20 miles, a forward speed of 5 mph, and a storm heading of -18 degrees from the north. Following Hall and Sobel^27^, this would create a storm with a 55° angle from perpendicular to the coastline with an annual frequency of 0.0034 with a return period of 294 years. Along the NJ coast, extreme Black Swan winds would generate much stronger surge and wave, and result in much higher structural loss and different wetland effects. At the Village of Piermont, the peak wind speed during the Black Swan storm would be stronger than the peak wind speed during Sandy, and the wind direction would be from the southeast instead of the east. This Black Swan wind in the Piermont region was used in a separate study to investigate the role of the Piermont marsh in protecting residential structures during storms.

#### Storm ensemble

Here, we consider a storm ensemble generated by Hall using the North Atlantic Stochastic Hurricane Model (NASHM)^44,45^. Then, we used JPM-OS to generate a set of optimal storms to represent all the possible storms described by Hall’s original storm ensemble^28,46^. Finally, we simulated the 1% annual chance flood and significant wave height with and without the wetlands and produced the corresponding maps according to the next equations:

4 $$ P\left[ {\eta_{max} > \eta } \right] = \lambda \smallint \cdots \smallint f_{x} \left( x \right)P\left[ {\eta \left( x \right) > \eta } \right]dx.{ } $$

Here, $\lambda$ is the mean annual rate for all storms on the site, $f_{x} \left( x \right)$ is the joint probability density function of the storm characteristics, and the conditional probability that a storm with certain characteristics $x$ will cause a water level height or significant wave height above $\eta$. This integral is evaluated for all possible combinations of storm characteristics. The integral in Eq. (4) is not easily determined and is usually approximated as a weighted summation of discrete storm parameters value^28,46,47^.

5 $$ P\left[ {\eta_{max} > \eta } \right] = \mathop \sum \limits_{i = 1}^{n} \lambda_{i} P[\eta \left( {x_{i} } \right) > \eta ]. $$

### Economic analysis

Following the procedure described in Loerzel et al.^17^, the economic analysis focused on the state of NJ for simplicity. To quantify the value of the coastal wetland for flood and wave protection, two simulations were performed, one with wetland and another without wetland. The without-wetland scenario was done by replacing Manning’s n values of the wetlands with 0.02, which represents the open water^13^. For the economic analysis, we identified the residential parcels that were flooded, as well as the inundation levels and significant wave heights of each parcel. The structure value and the number of stories at every parcel are needed to use the depth damage functions from USACE^21^.

The structural data used in this study included the residential parcels in the ‘Parcels and MOD-IV Composite of New Jersey’ shapefile from the New Jersey Office of Information Technology, Office of GIS (NJOGIS)^48^. Basements were unaccounted for due to lack of information. The value of the structure was assumed to be uniform on any single parcel. Following Kousky and Walls^49^, a partially inundated parcel was included in the economic analysis if and only if the parcel’s centroid is inside the inundation layer. The monetary value used in the analysis for each parcel was the “improvement value” reported in 2018 USD. The loss percent was calculated using the interpolated flood depth at each parcel centroid. Residential parcels without “improvement value” or building description in terms of the number of stories were removed from the analysis. The numbers of stories were rounded to the closest integer.

We combined flood and wave for economic analysis. First, the depth-limited controlling wave height is calculated as $H_{c} = \min \left( {0.78d,\; 1.6H_{sig} } \right)$ where $d$ is the flood, and $H_{sig}$ is the significant wave height. The next step is to determine if the structure is in A zone, Coastal A zone, or V zone, according to FEMA. A zone is defined as where $H_{c}$ is lower than 1.5 ft, Coastal A zone is where waves are between 1.5 ft and 3.0 ft, and V zone is where $H_{c}$ is higher than 3.0 ft^50^. Next, the total water depth above the first-floor elevation is calculated as $D = d + 0.7H_{c} - FFE$ for Coastal A and V zones and $D = d - FFE$ for A zone, where $FFE$ is the first-floor elevation. Then we used this newly updated flood elevation which accounted for the wave crest for the USACE NACCS damage functions. The wave damage functions were applied to structures in the V zone, and the flood damage functions were applied to structures in Coastal A and A zones. The structural loss of each parcel ($PL$) was calculated by multiplying the structure damaged by the parcel “improvement value”. Finally, for validations purpose only, we incorporated the maximum NFIP coverage for small structures by setting the transformed structure loss $PL_{T} = {\text{min}}\left( {PL,\$ 250,000} \right)$. The total residential structural loss was then computed by adding together the structural losses of each parcel ($PL$) in the same zip-code. Avoided losses for every zip-code were calculated by subtracting the structural losses of the wetland-absent case from that of the wetland-present case. We normalized the NFIP payouts to 2018 USD using the county housing units^51–53^ and data reported in Weinkle et al.^53^.

It should be noted that the surge and wave were calculated by process-based dynamically coupled surge-wave models and hence the simulated surge and wave contain nonlinear surge-wave interactions. On the other hand, the damage assessment was based on empirical engineering understanding of flood and wave impact on structures and used empirical formulations including empirically determined damage functions. In this study, we followed the approach of USACE and FEMA without reinventing their empirical methods and formulas, by using the 1% flood elevation and 1% wave height simulated by the coupled CH3D-SWAN model to better represent the wave effect on total flood depth and damage. While this may not be the perfect approach for damage assessment, our analysis provided a conservative damage estimation which probably slightly over-estimated the total damage by using the sum of maximum flood and maximum wave as the total maximum flood depth during each storm. Nevertheless, the good agreement between our assessed damage during Sandy and the NFIP payouts confirmed the robustness of our method. Further improvement of the empirical damage assessment may require additional data, model simulation, and parameter tuning which could be the topic of a future study.

## Supplementary Information

Supplementary Information.

## Data Availability

The topography, bathymetry, and land-use datasets used in the regional and local studies are available via the sources described above. The data on New Jersey structure value is available from https://njogis-newjersey.opendata.arcgis.com/datasets/406cf6860390467d9f328ed19daa359d.The NFIP payout data is available from https://www.fema.gov/media-library/assets/documents/180374. Damage functions are available from the U.S. Army Corps of Engineers (Physical Depth Damage Function Summary Report, North Atlantic Comprehensive Coastal Study: Resilient Adaptation to Increasing Risk). All derived data such as differences in losses and flood and wave heights between the two scenarios for the regional study, are available from the corresponding author on reasonable request and may be subject to a suitable Non-Disclosure Agreement.

## Code availability

CH3D and SWAN have both been described in detail in the literature cited. Any inquiry about these models should be directed to the corresponding author and TU-Delft, respectively. Wetland coverage data are available from the corresponding author upon reasonable request.
