# Supplementary material for: Role of wetlands in reducing structural loss is highly dependent on characteristics of storms and local wetland and structure conditions
Source: Sci Rep. 2021 Mar 4;11:5237. doi: 10.1038/s41598-021-84701-z (PMC7933150; doi:10.1038/s41598-021-84701-z)
Supplement: Supplementary file 1 — Supplementary Information. [file 41598_2021_84701_MOESM1_ESM.docx]

Role of Wetlands in Reducing Structure Damage is Highly Dependent on Storm Characteristics and Local Conditions

Y. Peter Sheng^1^*, Adail A. Rivera-Nieves^1^, Ruizhi Zou^1^, and Vladimir A. Paramygin^1^

1Coastal and Oceanographic Engineering Program, University of Florida, Gainesville, Florida, 32607, USA.

*Corresponding author: Y. Peter Sheng ([pete@coastal.ufl.edu)](mailto:pete@coastal.ufl.edu))

# SI Figures and Tables


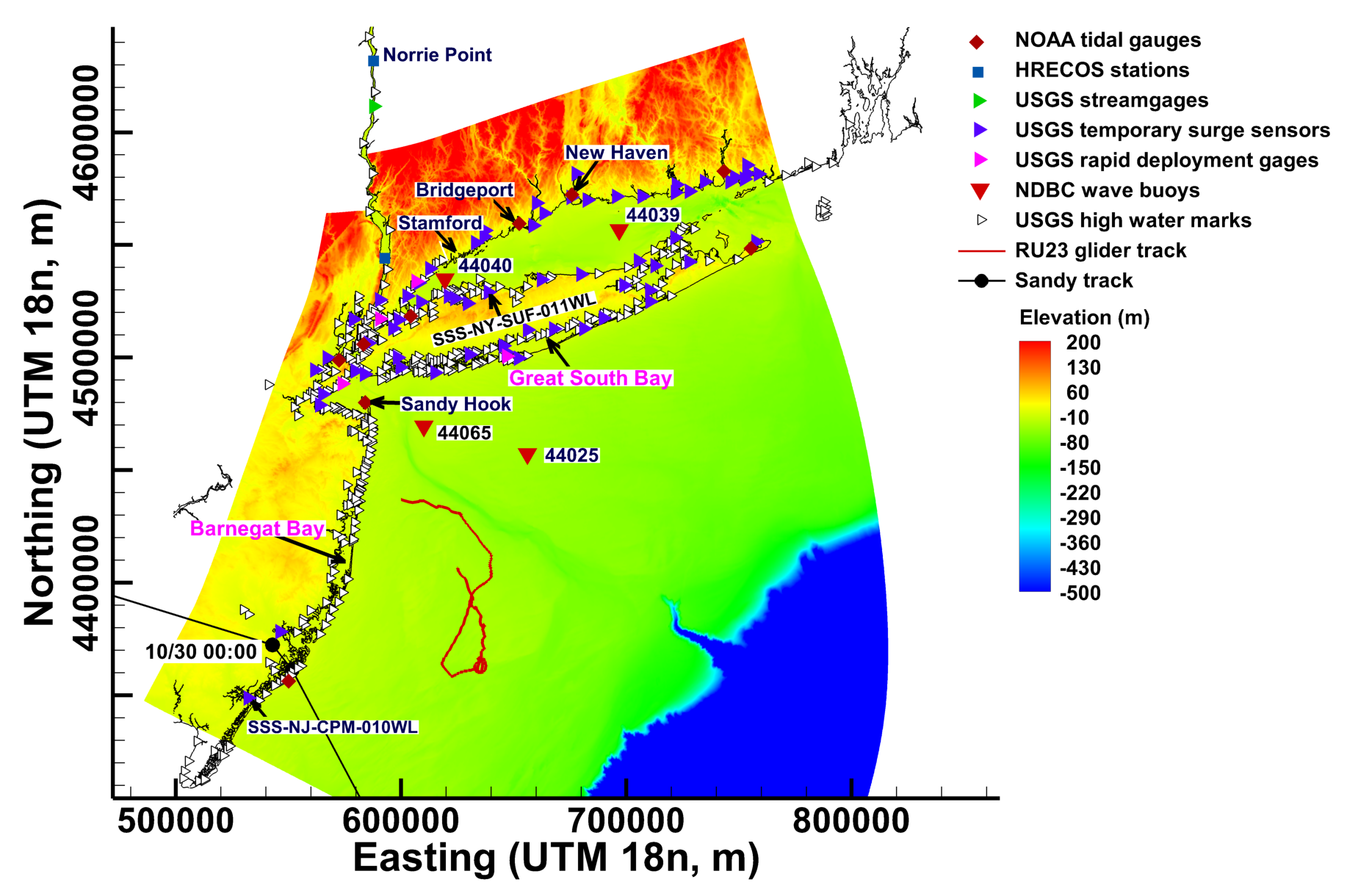


**Figure SI 1:** Grid domain covering the coasts of New Jersey (NJ), New York (NY), and Connecticut (CT). Hurricane Sandy's best track from the National Hurricane Center (NHC) is shown by a black solid line with black dots; the track of RU23 glider is depicted in a red solid line; the temporary and permanent gages, wave buoys, and high water marks are distributed over the domain. The map is produced using TecPlot 360 2020 (https://www.tecplot.com).


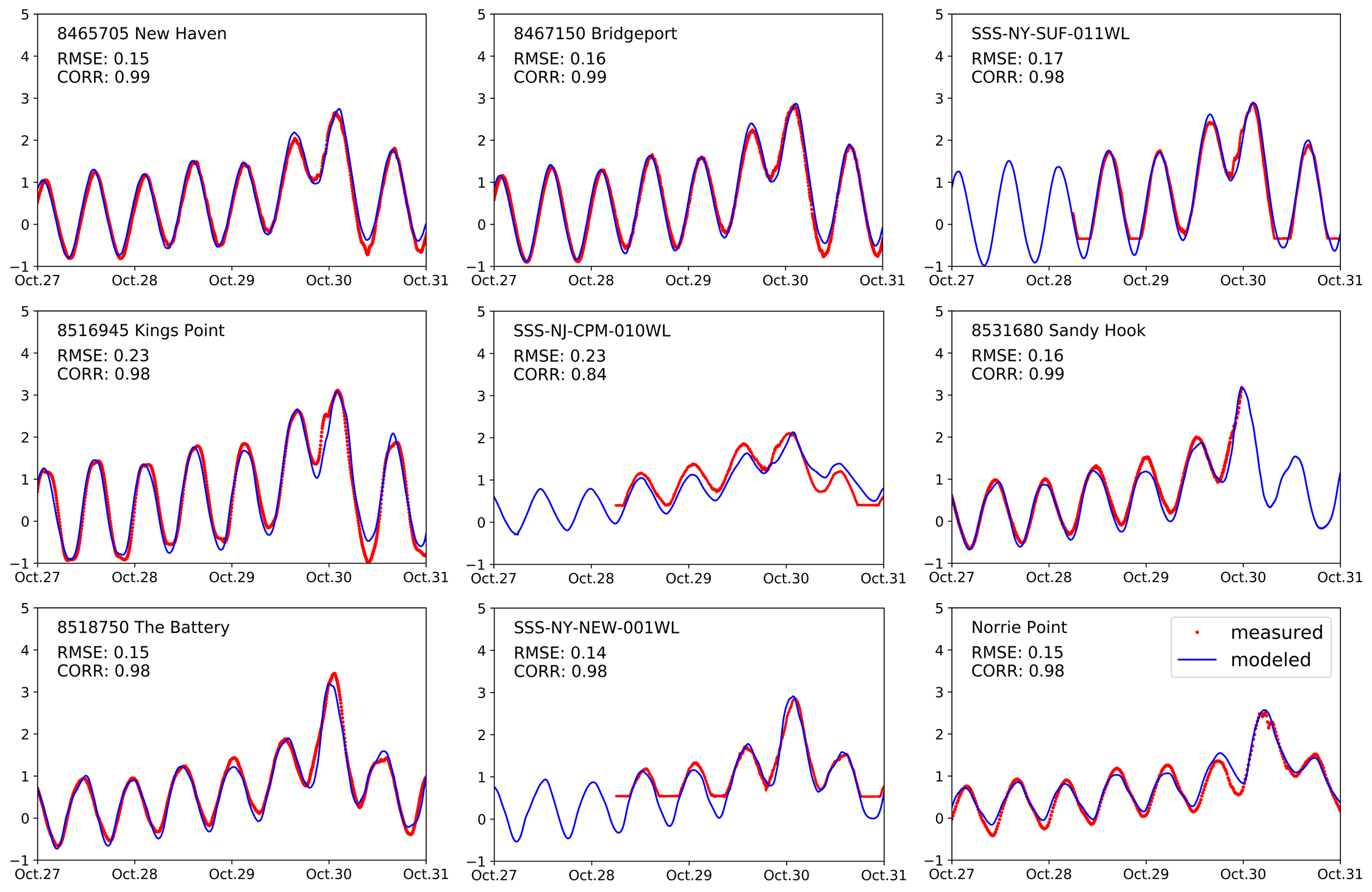


**Figure SI 2:** Flood model time-series validation for the scenario “With Wetlands” during Hurricane Sandy. Comparison of CH3D-SSMS model results (blue line) with the observed water levels (red dots) at the water level stations from NOAA^1^, HRECOS^2^, USGS^3^, the RMSE, and correlation coefficient (CORR) are given in each panel. The figure is produced using Python 3.9.1 (https://www.python.org/).


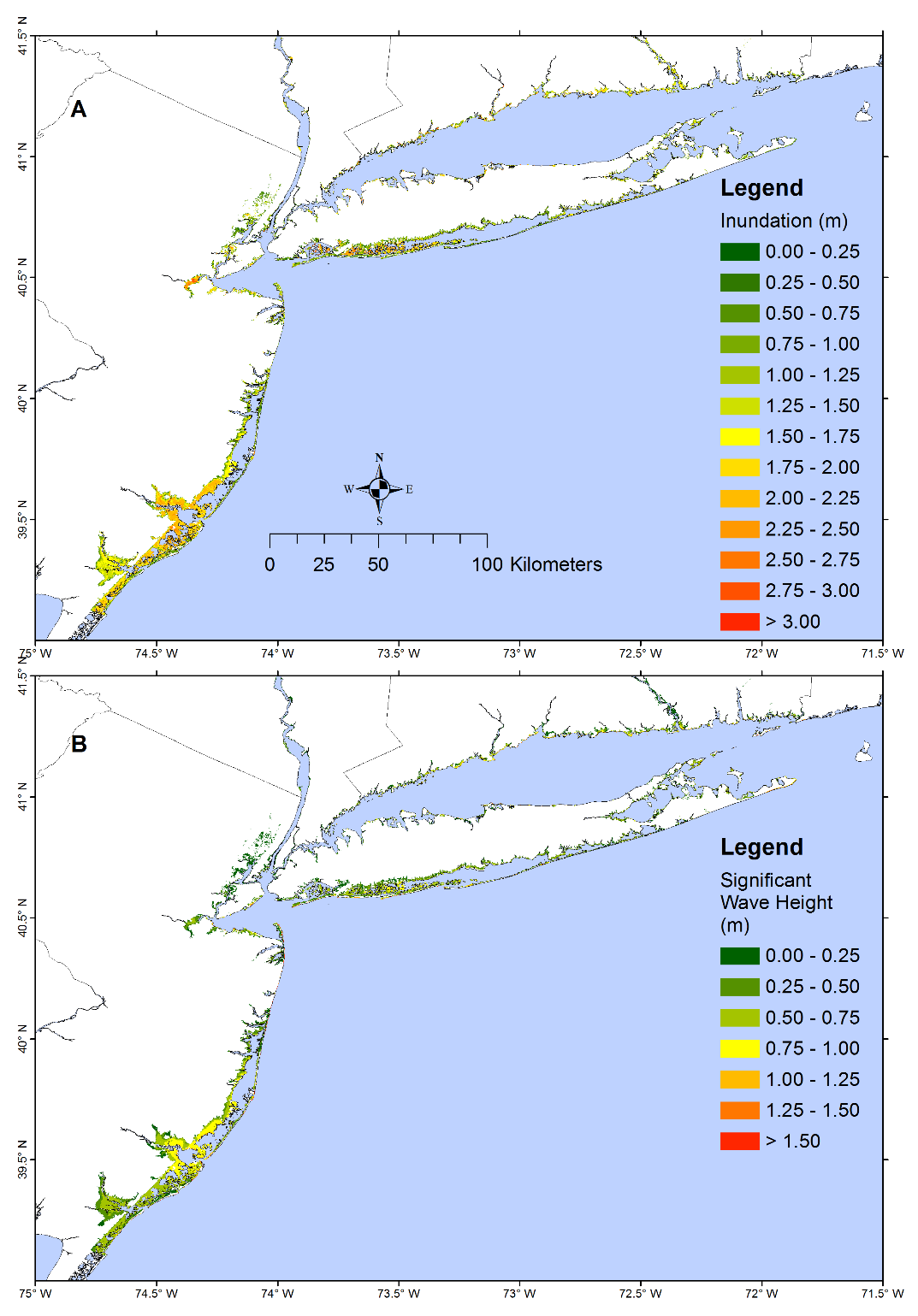


**Figure SI 3:** Model results for the “With Wetlands” scenario during Hurricane Sandy. A) Maximum inundation height and B) maximum significant wave height. The map is produced using ESRI ArcGIS Pro 2.7 (https://www.esri.com/en-us/arcgis/products/arcgis-pro/overview).





**Figure SI 4:** Flood model high water-marks validation for the “With Wetlands” scenario during Hurricane Sandy. Correlation of USGS high water-marks^3^ and CH3D-SSMS computed values. The figure is produced using Python 3.9.1 (https://www.python.org/).


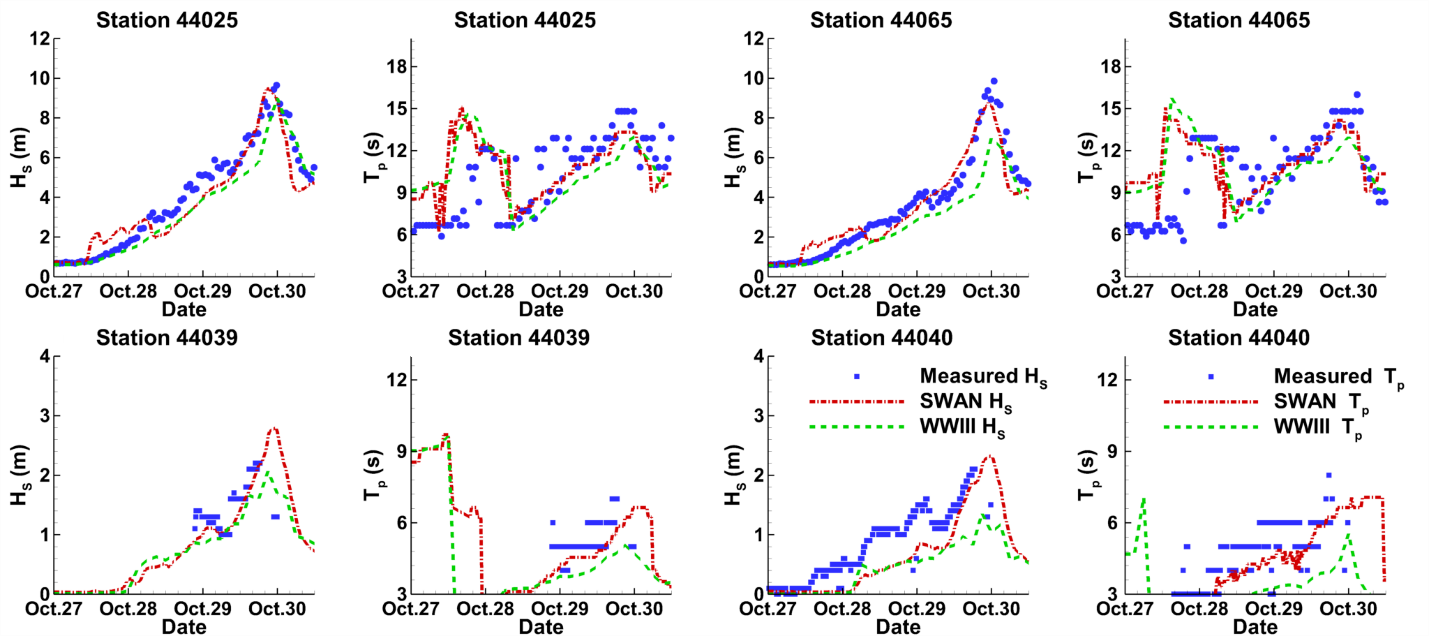


**Figure SI 5:** Wave Model validation for the “With Wetlands” scenario during Hurricane Sandy. Comparison of SWAN modeled results (red dash-dotted lines) with measured data^4^ (blue dots) and the results from NOAA’s operational WW3^5^ (green dashed lines). The figure is produced using Python 3.9.1 (https://www.python.org/).


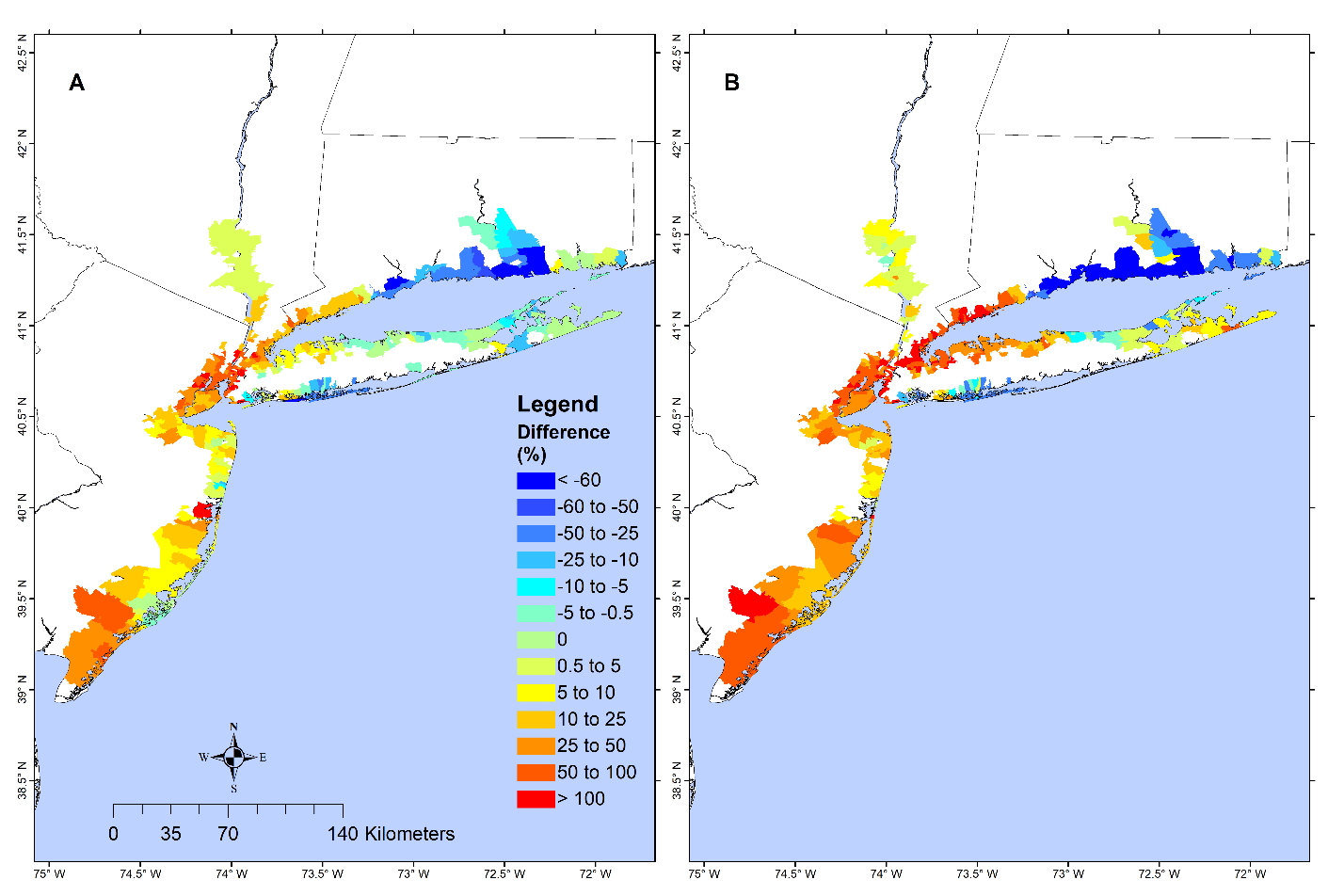


**Figure SI 6:** Zip-code resolution wetland’s effect on total inundation volume ($TIV$) and total wave energy ($TWE$) during the Black Swan storm. Maps showing zip-code resolution avoidance in A) $TIV$ and B) $TWE$ without wetlands, as a percentage of those for the with-wetland scenario. Dark red values show zip-code with the most wetland benefit while dark blue areas have the least wetland value. Negative values indicate that the presence of wetland would increase $TIV/TWE$ and positive values indicate that wetland would lower$TIV/TWE$. The map is produced using ESRI ArcGIS Pro 2.7 (https://www.esri.com/en-us/arcgis/products/arcgis-pro/overview).


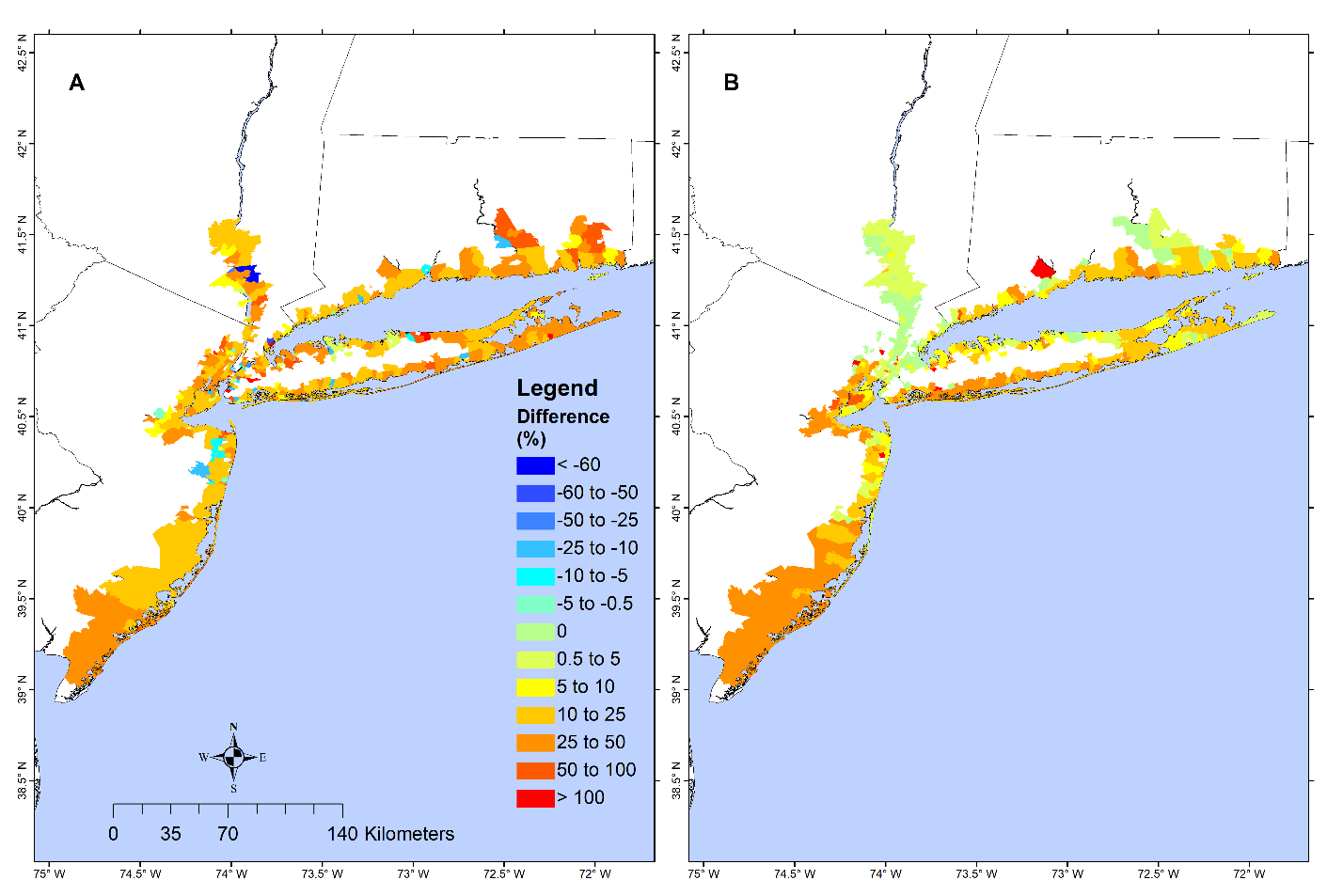


**Figure SI 7:** Zip-code effect of wetlands on $TIV$ and $TWE$ for the 1% annual chance events. Map showing zip code resolution difference in A) $TIV$ and B) $TWE$ if the wetlands were absent, as a percentage of those for the wetland present scenario. Dark red values show zip code with the most benefit of having wetlands while dark blue areas show the least benefited area. Negative values indicate that the presence of wetland would increase $TIV/TWE$ and positive values indicate that wetland would lower$TIV/TWE$. The map is produced using ESRI ArcGIS Pro 2.7 (https://www.esri.com/en-us/arcgis/products/arcgis-pro/overview).


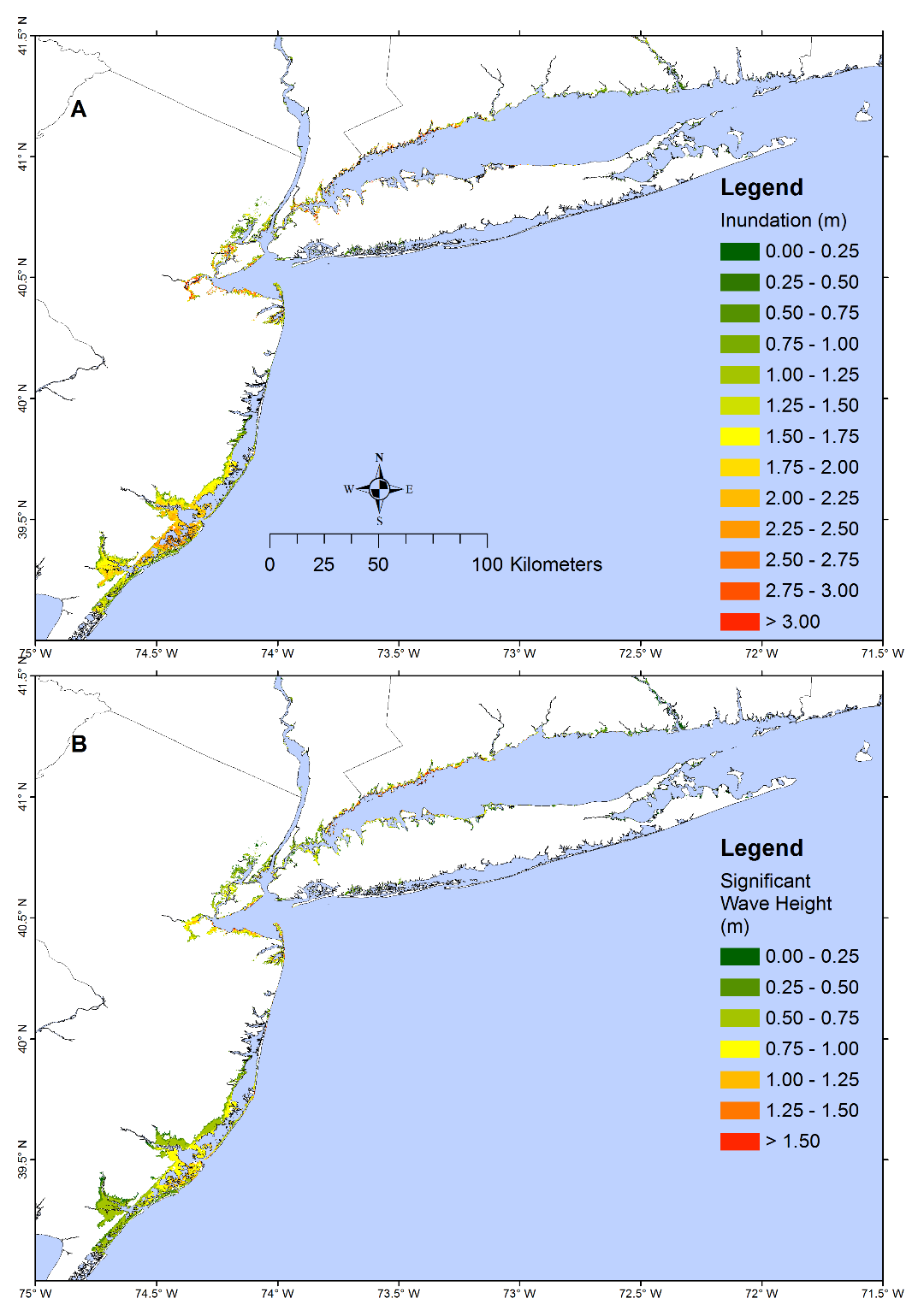


**Figure SI 8:** Model results for the “With Wetlands” scenario during the Black Swan storm. A) Maximum inundation height and B) maximum significant wave height. The map is produced using ESRI ArcGIS Pro 2.7 (https://www.esri.com/en-us/arcgis/products/arcgis-pro/overview).


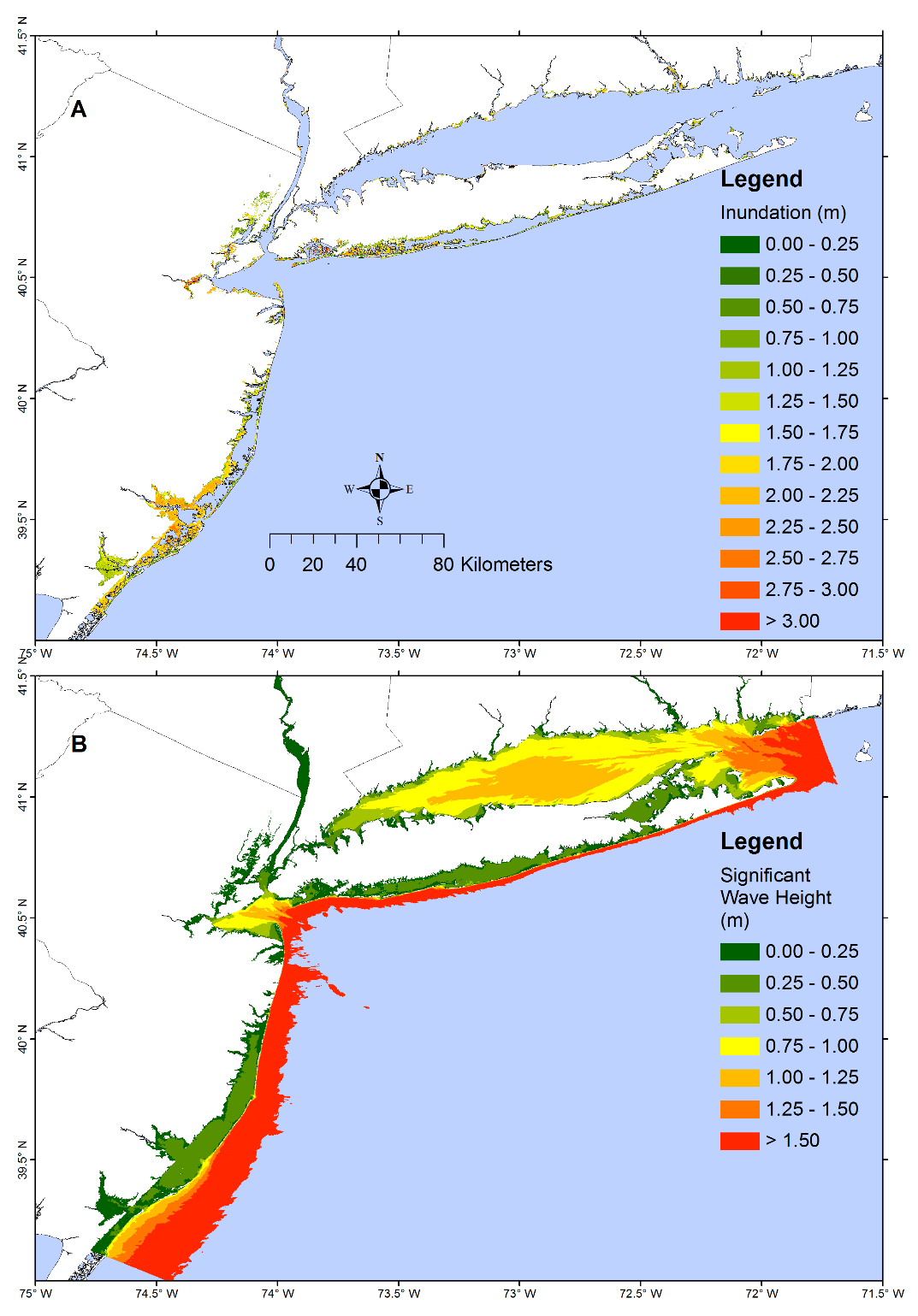


**Figure SI 9:** Model results for the “With Wetlands” scenario for the 1% annual chance events. A) Maximum inundation height and B) maximum significant wave height. The map is produced using ESRI ArcGIS Pro 2.7 (https://www.esri.com/en-us/arcgis/products/arcgis-pro/overview).


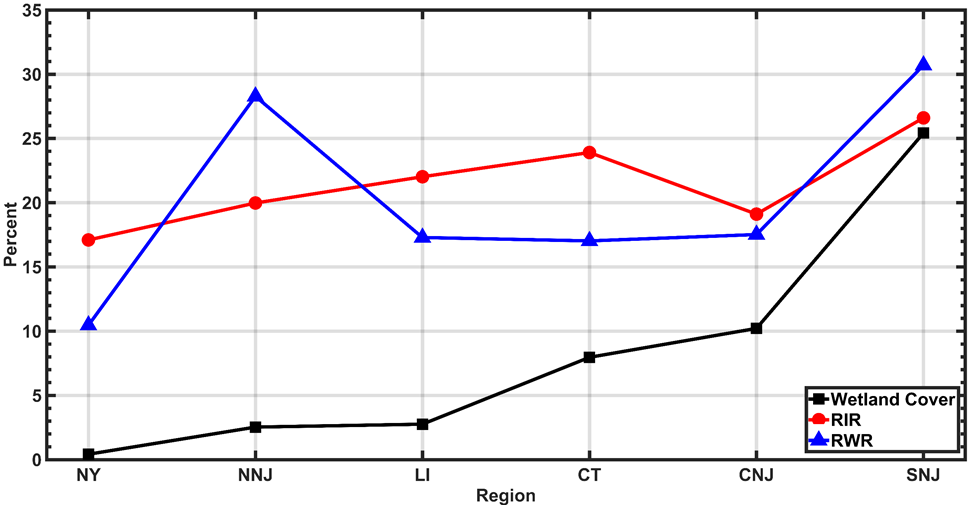


**Figure SI 10:** Percent wetland cover, RIR (relative TIV reduction), and RWR (relative wave energy reduction) in six regions (New York, North New Jersey, Long Island, Connecticut, Central New Jersey, and South New Jersey) during 1% annual chance events. As the wetland cover increases from less than 5% (NY, NNJ, and LI) to more than 10% in CNJ and SNJ, RIR and RWR generally increase, showing the increasing role of wetland in reducing inundation and wave. Relative reduction in inundation and wave energy are modest, between 10% and 30%. NNJ has properties behind the relatively sparse marsh, followed by woody wetland which protects properties behind them. Connecticut has less wetland than Central Jersey, but the mostly woody wetland is more effective in reducing flood and wave. The figure is produced using Matlab R2020 (https://www.mathworks.com/).


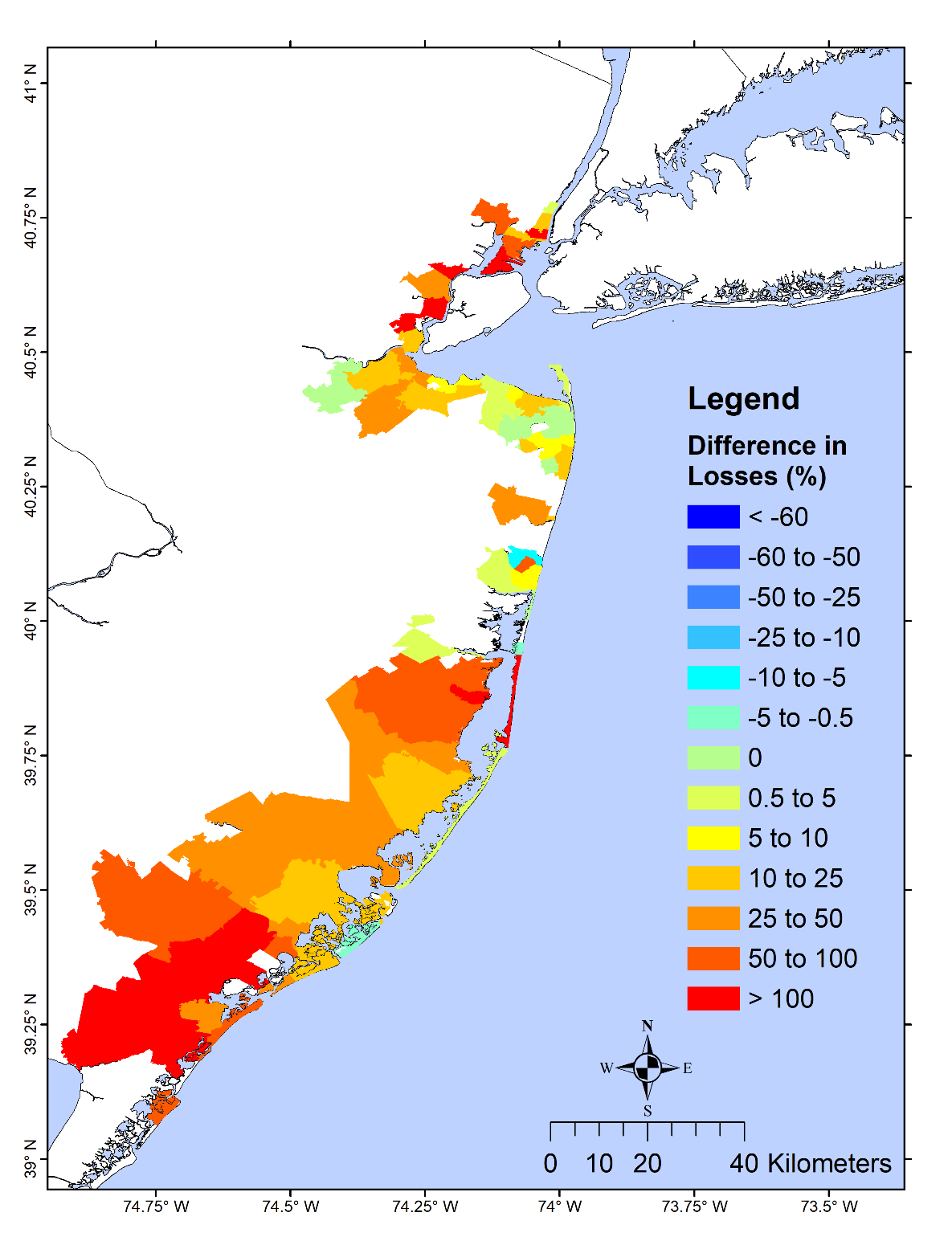


**Figure SI 11:** Effect of wetlands on structure damage over zip code scale during the Black Swan storm. Map showing zip code resolution difference in losses if the wetlands were absent, as a percentage of the wetland-present scenario. Dark red values show zip code with the highest benefit of having wetlands while dark blue areas show the least benefited area. Negative values indicate that the presence of wetland would increase structure losses and positive values indicate that wetland would lower the structure losses. The map is produced using ESRI ArcGIS Pro 2.7 (https://www.esri.com/en-us/arcgis/products/arcgis-pro/overview).


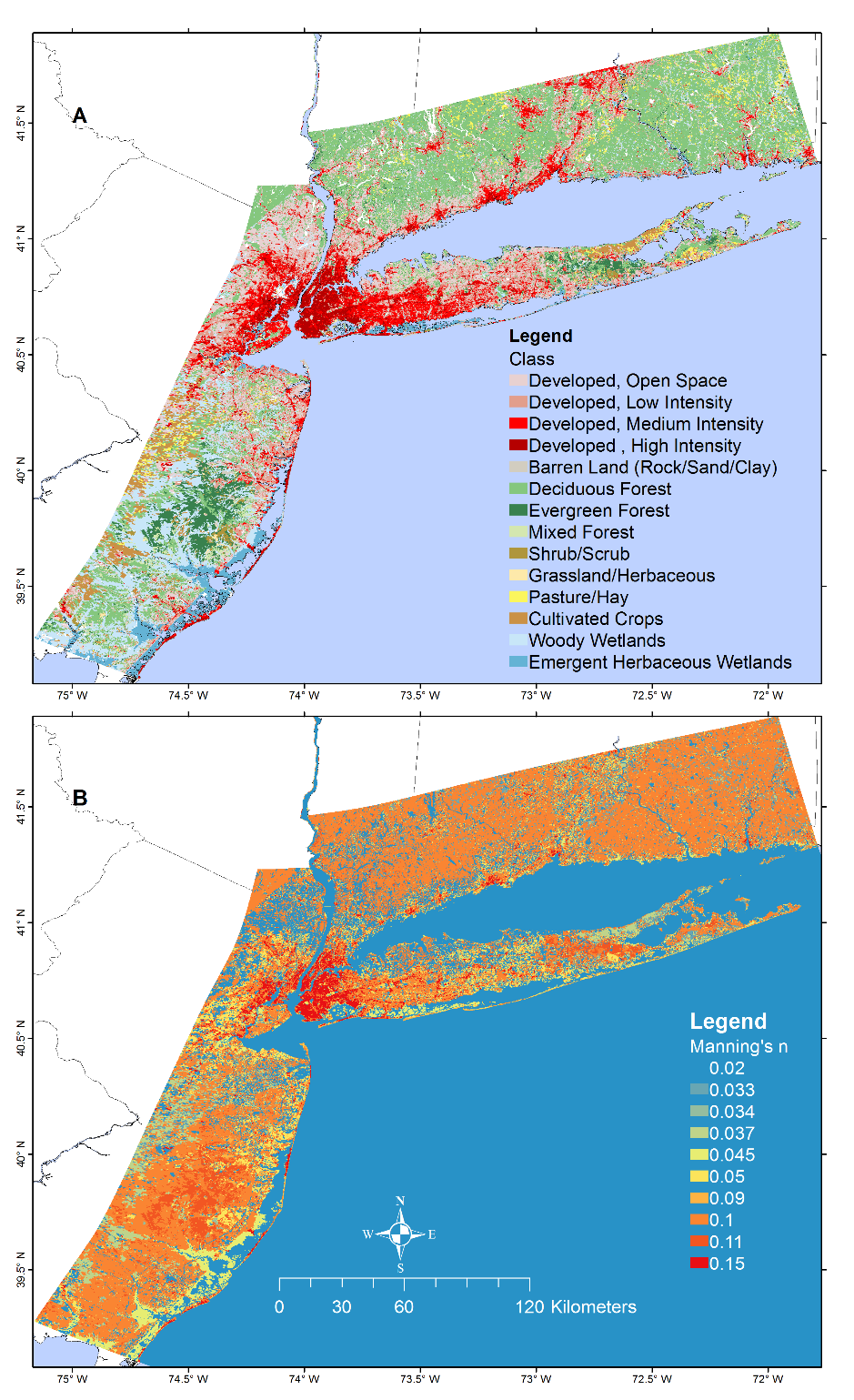


**Figure SI 12:** Landcover (A) and Manning’s n coefficient (B) maps. Land Cover types were obtained from the USGS 2011 National Land Cover Database6. Manning’s n coefficient is based on the conversion table from Mattocks and Forbes (2008)^7^. The map is produced using ESRI ArcGIS Pro 2.7 (https://www.esri.com/en-us/arcgis/products/arcgis-pro/overview).


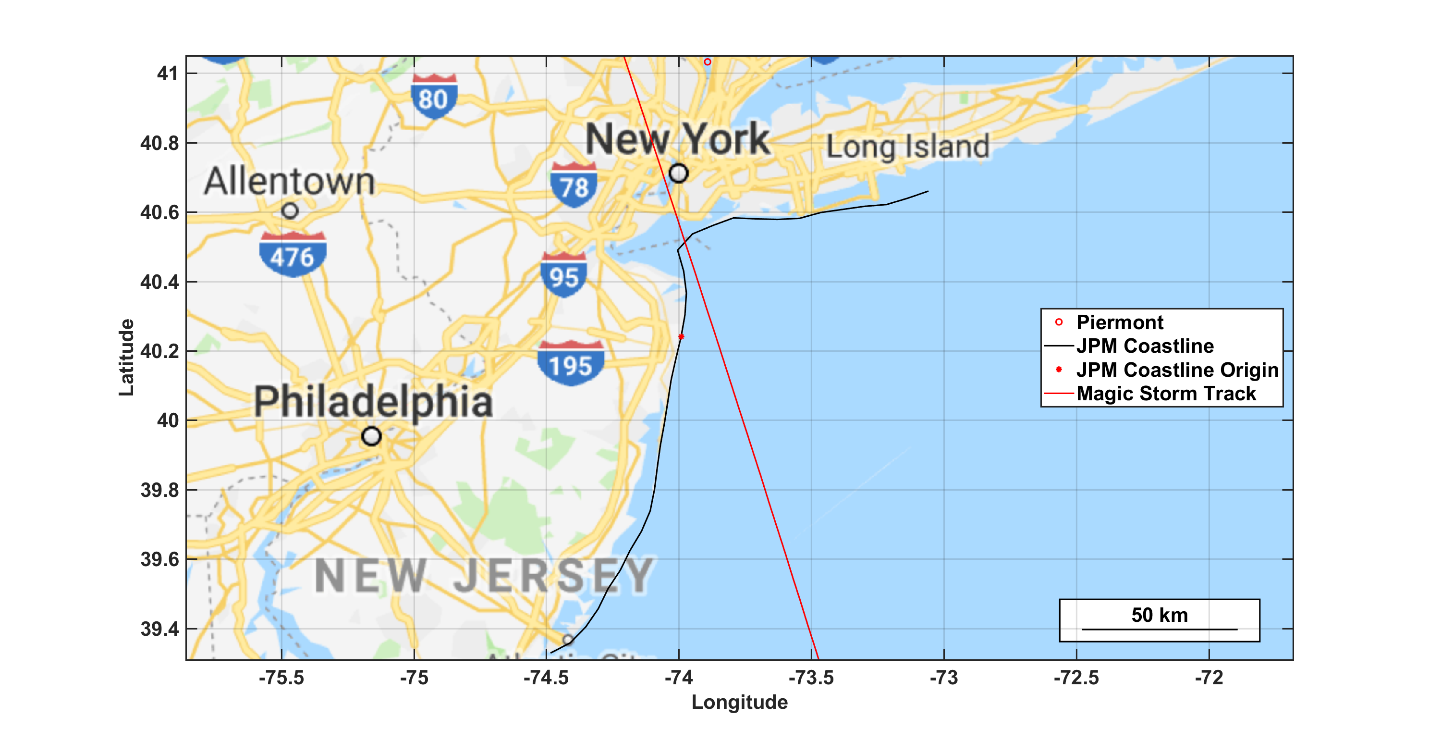


**Figure SI 13:** Black Swan storm track. Redline represents the Black Swan storm track. The coastline used for the storm ensemble JPM-OS is represented as the black line with its origin represented as a red filled circle. The map is produced using Matlab R2020 (https://www.mathworks.com/). Base map: Google Maps.

**Table SI 1:** Flood model validation Summary. Averaged RMSE and CORR for the modeled storm tides at stations from NOAA^1^, HRECOS^2^, and USGS^3^.

| Data source | Station number | Average RMSE (m) | Average CORR |
| --- | --- | --- | --- |
| NOAA tidal gauges | 9 | 0.17 | 0.98 |
| HRECOS stations | 2 | 0.20 | 0.96 |
| USGS stream gages | 1 | 0.14 | 0.97 |
| USGS temporary storm tide sensors | 61 | 0.20 | 0.94 |
| USGS rapid deployment gages | 4 | 0.21 | 0.96 |

**Table SI 2:** Regional values of relative inundation reduction and relative wave reduction. RIR is the difference between TIV for the no-wetland case and the TIV for the with-wetland case, as a percent of the with-wetland TIV. RWR is the difference between TWE for the no-wetland case and the TIV for the with-wetland case, as a percent of the with-wetland TIV. Relative inundation and wave reduction depend significantly on the specific storm scenario and wetland conditions in each region (see **Table SI 4**). South New Jersey has the highest relative flood and wave reduction during Sandy and 1% event, while NY has the highest reduction during the BS storm.

| Case | Region | Relative Reduction of Inundation metrics | | | | Relative Reduction of  Wave metrics | | |
| --- | --- | --- | --- | --- | --- | --- | --- | --- |
|  |  | AIH | MIH | TIA | TIV | AWH | MWH | TWE |
| Sandy | New York | 0.08% | -0.30% | 0.11% | 0.19% | 1.64% | 1.65% | 3.90% |
|  | Long Island | 0.93% | 0.00% | 1.55% | 2.49% | 3.98% | 10.86% | 9.91% |
|  | North Jersey | 0.69% | 0.00% | 5.09% | 5.82% | -3.47% | 0.90% | 3.77% |
|  | Central Jersey | 2.43% | -0.32% | 1.95% | 4.43% | 9.24% | -0.81% | 20.10% |
|  | South Jersey | 0.94% | 7.41% | 3.38% | 4.35% | 10.81% | 4.83% | 25.49% |
|  | Connecticut | 0.78% | 0.32% | 0.46% | 1.24% | 3.78% | -0.13% | 6.58% |
| Black Swan | New York | 13.22% | 7.33% | 15.57% | 30.85% | 28.45% | 15.74% | 79.06% |
|  | Long Island | -0.11% | -0.70% | 8.73% | 8.61% | 22.69% | 4.80% | 54.37% |
|  | North Jersey | 8.83% | 7.32% | 24.01% | 34.95% | 11.31% | 2.48% | 51.65% |
|  | Central Jersey | 4.59% | 4.21% | 4.67% | 9.47% | 12.78% | 3.22% | 31.51% |
|  | South Jersey | 13.19% | 11.19% | 6.14% | 20.14% | 13.90% | 3.08% | 34.71% |
|  | Connecticut | 18.81% | 7.01% | -13.24% | 3.08% | 36.09% | 11.83% | 56.39% |
| 1% Annual Chance Flood | New York | 17.15% | 5.58% | -0.04% | 17.10% | 7.79% | 6.44% | 10.46% |
|  | Long Island | 23.60% | 5.65% | -1.28% | 22.02% | 10.26% | 14.67% | 17.29% |
|  | North Jersey | 21.40% | 5.11% | -1.18% | 19.98% | 17.81% | 3.23% | 28.28% |
|  | Central Jersey | 19.88% | 6.05% | -0.64% | 19.11% | 11.47% | 9.06% | 17.52% |
|  | South Jersey | 28.10% | 13.22% | -1.17% | 26.60% | 15.43% | 0.00% | 30.70% |
|  | Connecticut | 25.85% | 8.39% | -1.55% | 23.91% | 9.63% | 0.00% | 17.03% |

**Table SI 3:** NJ wetlands effect on structural damage. Wetland effect on New Jersey structure losses during Sandy, Black Swan storm, and 1% flood for the scenarios with wetland and without wetland. The last column shows the increase in losses as a percentage of the with-wetland scenario. Sandy (NFIP) use transformed structure loss ($\boldsymbol{P}\boldsymbol{L}_{\boldsymbol{T}}$) all other cases use the structure loss ($\boldsymbol{PL}$). Values inside parenthesis represent the percentage of total damage.

| Storm | Variable | With Wetlands | Without Wetlands | Difference ($) | Percent |
| --- | --- | --- | --- | --- | --- |
| Sandy (NFIP, USD) | Flood | 3,138,251,788  (86.76%) | 3,302,065,065  (84.08%) | 163,813,277 | 5.22% |
|  | Wave | 478,715,087  (13.24%) | 625,109,836  (15.92%) | 146,394,749 | 30.58% |
|  | Total | 3,616,966,875 | 3,927,174,901 | 310,208,026 | 8.58% |
| Sandy (USD) | Flood | 3,372,696,568  (83.04%) | 3,540,471,545  (80.37%) | 167,774,977 | 4.97% |
|  | Wave | 688,730,809  (16.96%) | 864,931,481  (19.63%) | 176,200,672 | 25.58% |
|  | Total | 4,061,427,377 | 4,405,403,026 | 343,975,649 | 8.47% |
| Black Swan (USD) | Flood | 3,202,796,673  (39.80%) | 4,114,281,862  (40.56%) | 911,485,189 | 28.46% |
|  | Wave | 4,843,714,608  (60.20%) | 6,030,226,313  (59.44%) | 1,186,511,705 | 24.50% |
|  | Total | 8,046,511,281 | 10,144,508,175 | 2,097,996,894 | 26.07% |
| 1% Annual Chance (Annual Loss, USD) | Flood | 30,978,597  (97.72%) | 47,111,365  (97.59%) | 16,132,769 | 52.08% |
|  | Wave | 723,233  (2.28%) | 1,165,418  (2.41%) | 442,185 | 61.14% |
|  | Total | 31,701,829 | 48,276,783 | 16,574,954 | 52.28% |

**Table SI 4:** Wetland cover of the study area. Emergent herbaceous wetlands are characterized by marshes (tidal wetlands) and wet meadows (freshwater wetlands). Red maple swamps and hemlock swamps are the typical woody wetland (freshwater wetlands) communities in NY and LI^8^. Woody wetlands are characterized by having plant heights greater than 6 meters tall in contrast to emergent herbaceous wetlands which are typically less than 1 meter of height in NJ. A shown in the table, NY, CT, and north NJ have little wetland coverage, while the south and central NJ have the highest wetland coverage. CT has less than 10% wetland area but most (>80%) of them are tall woody wetlands.

| Region | Emergent Herbaceous Cover % | Woody Wetland % | Wetlands % |
| --- | --- | --- | --- |
| NY | 0.01% | 0.41% | 0.42% |
| NNJ | 0.36% | 2.18% | 2.53% |
| LI | 2.22% | 0.54% | 2.76% |
| CT | 1.20% | 6.76% | 7.97% |
| CNJ | 1.18% | 9.03% | 10.22% |
| SNJ | 9.03% | 16.40% | 25.42% |

# SI References

1. National Oceanic and Atmospheric Administration (NOAA). NOAA Tides and Currents. https://tidesandcurrents.noaa.gov/.

2. Hudson River Environmental Conditions Observing System (HRECOS). Historical Data. https://hrecos.org/index.php?option=com_content&view=article&id=143&Itemid=54.

3. United States Geological Survey (USGS). Flood Event Viewer. https://stn.wim.usgs.gov/FEV/#Sandy.

4. National Oceanic and Atmospheric Administration (NOAA). National Data Buoy Center. https://www.ndbc.noaa.gov/.

5. The WAVEWATCH III Development Group (WW3DG). *User manual and system documentation of WAVEWATCH III version 6.07 Tech. Note 333, NOAA/NWS/NCEP/MMAB*. (2019).

6. Homer, C. G. *et al.* Completion of the 2011 National Land Cover Database for the conterminous United States – Representing a decade of land cover change information. *Photogramm. Eng. Remote Sensing* **81**, 345–354 (2015).

7. Mattocks, C. & Forbes, C. A real-time, event-triggered storm surge forecasting system for the state of North Carolina. *Ocean Model.* **25**, 95–119 (2008).

8. NYCDEP. Wetlands in the Watersheds of the New York City Water Supply System. *Wetlands*.
